# Iron Oxide Nanoparticle-Induced Autophagic Flux Is Regulated by Interplay between p53-mTOR Axis and Bcl-2 Signaling in Hepatic Cells

**DOI:** 10.3390/cells9041015

**Published:** 2020-04-18

**Authors:** Mariia Uzhytchak, Barbora Smolková, Mariia Lunova, Milan Jirsa, Adam Frtús, Šárka Kubinová, Alexandr Dejneka, Oleg Lunov

**Affiliations:** 1 Institute of Physics of the Czech Academy of Sciences, 18221 Prague, Czech Republic; uzhytchak@fzu.cz (M.U.); smolkova@fzu.cz (B.S.); mariialunova@googlemail.com (M.L.); frtus@fzu.cz (A.F.); sarka.kubinova@iem.cas.cz (Š.K.); dejneka@fzu.cz (A.D.); 2 Institute for Clinical & Experimental Medicine (IKEM), 14021 Prague, Czech Republic; miji@ikem.cz; 3 Institute of Experimental Medicine of the Czech Academy of Sciences, 14220 Prague, Czech Republic

**Keywords:** nano-bio interactions, iron oxide nanoparticles, autophagy, lysosomes, magnetic resonance imaging, p53

## Abstract

Iron oxide-based nanoparticles have been repeatedly shown to affect lysosomal-mediated signaling. Recently, nanoparticles have demonstrated an ability to modulate autophagic flux via lysosome-dependent signaling. However, the precise underlying mechanisms of such modulation as well as the impact of cellular genetic background remain enigmatic. In this study, we investigated how lysosomal-mediated signaling is affected by iron oxide nanoparticle uptake in three distinct hepatic cell lines. We found that nanoparticle-induced lysosomal dysfunction alters sub-cellular localization of pmTOR and p53 proteins. Our data indicate that alterations in the sub-cellular localization of p53 protein induced by nanoparticle greatly affect the autophagic flux. We found that cells with high levels of Bcl-2 are insensitive to autophagy initiated by nanoparticles. Altogether, our data identify lysosomes as a central hub that control nanoparticle-mediated responses in hepatic cells. Our results provide an important fundamental background for the future development of targeted nanoparticle-based therapies.

## 1. Introduction

Despite decades of research, iron oxide-based nanoparticles (NPs) still attract researchers’ attention in different areas of biomedical studies [1,2,3]. Iron oxide NPs have been successfully implemented in widespread biomedical applications, including targeted drug delivery, imaging, biosensors, and different therapeutic modalities [1,2,3,4,5,6,7]. The application of iron oxide-based NPs as magnetic resonance imaging (MRI) contrast agents initially found a way to clinical approval [8,9]. However, such contrast agents have been withdrawn or discontinued due to various side effects [8,9,10,11], clearly indicating their overlooked cytotoxic potential. Indeed, studies revealed that oxidative stress due to excessive reactive oxygen species (ROS) accumulation is a source of iron oxide NP-triggered cytotoxicity [12,13,14,15,16]. Furthermore, more recent studies have shown that certain NP concentrations can induce significant cellular responses without noticeable oxidative stress [17]. In fact, we have already reported the evidence that subcytotoxic doses of NPs may induce alteration in subcellular signaling [18]. However, revealing the precise molecular mechanisms of such actions of nanoparticles requires more intensive studies.

A growing body of evidence has identified the liver as a predominant end point of the majority of intravenously administered nanoparticles—for review see [19]. In fact, particles with diameter of ~ 150–200 nm have been shown to penetrate the space of Disse and directly interact with hepatocytes [19,20]. Such an direct interaction of particles with hepatocytes allows them to be excreted from the body via the hepatobiliary pathway [19]. Surprisingly, reports about responses of hepatic cells to NP sub-lethal treatment are limited [19]. In fact, a direct comparison of the observed effects on closely related cell lines is lacking [16,19,20].

It becomes evident that dextran decoration of iron oxide NPs shell results in predominant endocytic uptake of NPs, especially if cells are unequipped for phagocytosis [21,22,23]. In addition, such nanoparticles will eventually reach lysosomes via the endo-lysosomal pathway [24]. It is worth noting here that lysosomal membranes are important regulators of the activity of mammalian target of rapamycin (mTOR), a key kinase that controls cell growth and proliferation [25]. In turn, mTOR actively participates in autophagy regulation and nutrient uptake control [18,26]. All these studies clearly indicate that lysosomes play a role of central hub in nutrient sensing, transcriptional regulation, and metabolic homeostasis within the cell [27]. It is important to add that a number of studies demonstrated that NPs can modulate mTOR activity [18]. Moreover, iron oxide NPs modulate autophagic flux [28,29]. Collectively, all these studies support that iron oxide NPs regulate cellular signaling via lysosomal-based modulation of mTOR activity. However, the specific role and underlying mechanism of lysosomal-induced signaling, especially in hepatic cells, are still poorly understood. Moreover, there is a lack of studies showing how the genetic background of different cells from a similar lineage may affect resultant signaling.

Therefore, in this study, we investigated in detail the signaling that originates from lysosomes upon iron oxide NPs uptake in hepatic cell lines and defined contribution of cellular genetic background in these processes. Our findings provide a holistic analysis of key proteins that contribute to lysosomal-mediated signaling in distinct hepatic cell lines.

## 2. Materials and Methods

### 2.1. Chemicals and Antibodies

All chemicals and antibodies used for this study are compiled in Tables S1 and S2 of Supplemental Materials including manufacturers, catalogue numbers and dilutions.

### 2.2. Cell Culture

Human hepatoblastoma HepG2 cell line (American Type Culture Collection, ATCC, Manassas, VA, USA), and human hepatocellular carcinoma cell lines Alexander (PLC/PRF/5, ATCC) and Huh7 (Japanese Collection of Research Bioresources, JCRB, Tokyo, Japan) were used in this study. Cells were cultured in EMEM medium (ATCC) supplemented with 10% fetal bovine serum (FBS, Thermo Fisher Scientific, Waltham, MA, USA) and 1% Penicillin/Streptomycin (Thermo Fisher Scientific). Cell cultures were cultivated in a humidified 5% CO2 atmosphere at 37 °C. Cell culture medium was replaced once a week.

### 2.3. Nanoparticles

In this study we used core-shell iron oxide NPs purchased from Chemicell (Chemicell GmbH, Berlin, Germany). The particles consist of a magnetite core coated with carboxymethyldextran shell. The surface can be modified with different kinds of fluorescent dyes allowing their detection using fluorescent microscopic techniques. We purchased green fluorescent (Ex: 476 nm, Em: 518 nm) labelled magnetic particles nano-screenMAG-CMX and non-fluorescent magnetic particles fluidMAG-CMX. Both types of NPs have 200 ± 20 nm (mean ± SD) the hydrodynamic diameter and similar physicochemical characteristics. Thorough physicochemical characterization of the particles has been performed previously in a number of publications [7,30,31,32,33]. Here, we utilize them solely as a NP model. The average size and zeta potential of the particles were measured using a Zetasizer Nano (Malvern Instruments, Malvern, UK).

### 2.4. Nanoparticle-Protein Interaction

Nanoparticles (5, 50 and 100 μg Fe mL^−1^) were incubated either in PBS, or in EMEM medium (ATCC) supplemented with 10% fetal bovine serum (FBS, Thermo Fisher Scientific) for 2 h at 37 °C. The particles were collected by strong NdFeB magnet and washed extensively with PBS. The proteins associated with the particles were eluted and denaturated in sample loading buffer and separated by gel electrophoresis. As control, EMEM medium supplemented with 10% fetal bovine serum was used. Gels were stained with Coomassie blue (AppliChem, Darmstadt, Germany).

### 2.5. Cell Viability Assay

Viability of the cells was analysed using well-established alamarBlue viability assay (Thermo Fisher Scientific). AlamarBlue assay is based on the cleavage of resazurin to resorufin by viable cells, which results into the increasing of overall fluorescence intensity. This fact allows accurate fluorometric quantification of the percentage of metabolically active cells in the culture. We performed alamarBlue viability assay in accordance with the manufacturer’s instructions and our verified treatment protocol [34]. Briefly, cells were seeded onto 96-well plates at the density of 5000 cells per well and treated with different concentration of nanoparticles for 24 h. After the treatment, alamarBlue reagent was added to each sample and incubated for 2 h at 37 °C. Fluorescence intensity (using excitation between 530 and 560 nm and emission at 590 nm) was measured using a TECAN microplate reader SpectraFluor Plus (TECAN, Mannedorf, Switzerland). Readings were done in quadruplicates; three independent experiments were performed for each measurement. Additionally, we performed a crosscheck to ensure that NPs themselves do not interfere with the assay reagent (data not shown).

### 2.6. Lipid Peroxidation Assay

Detection of lipid peroxidation upon nanoparticle treatment was done using BODIPY™ 581/591 C11 lipid peroxidation sensor (Thermo Fisher Scientific). The fluorescent probe is intrinsically lipophilic and undergoes native-like transport and metabolism in cells [35]. Upon oxidation the BODIPY™ 581/591 C11 exhibit a shift of the fluorescence emission peak from ~590 nm to ~510 nm, resulting in decrease of red fluorescence [36,37]. Thus, the intensity of red fluorescence can be used as an indicator of lipid peroxidation under oxidative stress [38,39]. We performed a lipid peroxidation assay in accordance with the manufacturer’s instructions and previously published protocols [38,39]. Briefly, cells were seeded onto 96-well clean bottom plates at the density of 5000 cells per well and treated with different concentration of nanoparticles for 24 h. Cells were incubated with the lipid peroxidation sensor at concentration of 1 µM for 30 min at 37 °C. Fluorescence intensity (Ex: 540 nm; Em: 595 nm) was measured by a TECAN microplate reader SpectraFluor Plus (TECAN, Mannedorf, Switzerland). Readings were done in quadruplicates. Three independent experiments were performed for each measurement. Normalized fluorescence data are presented as means ± SEM. Additionally, we performed a crosscheck that NPs themselves do not interfere with the assay reagent (data not shown).

### 2.7. Analysis of Nanoparticles Uptake Kinetics

We assessed NPs uptake kinetics by fluorometric quantification. Cells onto 96-well clean bottom plates at the density of 5000 cells per well and treated with different concentration of fluorescently-labelled NPs for 2, 6, 8, and 24 h. Afterwards, cells were washed with PBS three times and incubated with 0.1% TrypanBlue in order to quench the fluorescent signal of NPs attached to the cell surface. TrypanBlue is widely used as a quencher of FITC fluorescence and is excluded from viable cells [40,41,42]. The NPs uptake was assessed by measuring of fluorescent intensity (Ex: 485 nm; Em: 540 nm) using TECAN microplate reader SpectraFluor Plus (TECAN, Mannedorf, Switzerland). Readings were done in quadruplicates; three independent experiments were performed for each measurement.

### 2.8. Immunofluorescence

Immunofluorescence staining was used in order to monitor NP subcellular localization, cytoskeleton dynamics and assessment of intracellular signaling events. Cells were seeded in 6-channel µ-Slides (Ibidi, Martinsried, Germany) at density 15,000 cells per well. Afterwards, cells were treated with different NP concentrations for indicated periods of time. After the treatment cells were washed with PBS and fixed with 4% paraformaldehyde in PBS pH 7.4 at room temperature for 10 min. Samples were permeabilized with 0.5% Triton X-100 before the staining. Immunofluorescence staining was performed on fixed cells using primary antibodies against different proteins summarized in Supplementary Table S2 and AlexaFluor 568- or AlexaFlour 488-conjugated secondary antibodies. Dilutions and catalogue numbers of used primary antibodies are given in Supplementary Table S2. Stained cells were imaged using spinning disk confocal microscopy IXplore SpinSR (Olympus, Tokyo, Japan). ImageJ software (NIH) was used for image processing.

### 2.9. Lysosomal Integrity Assay

Cells were seeded onto 96-well clear bottom plates (BD Biosciences, Prague, Czech) at a density of 5000 cells per well. After cells were incubated with cell culture media (EMEM, 10% FBS) containing different concentrations of nanoparticles for 24 h. For lysosomal stability assessment, we utilized an acridine orange (AO) assay. The AO assay was performed in accordance with our previously verified protocol [6,34]. Briefly, cells with incorporated nanoparticles were labeled with 5 µg mL^−1^ AO in culture medium for 15 min at 37 °C. Following nanoparticle treatment, cells were cultured at 37 °C for indicated periods of time and the intensity of orange fluorescence was then measured using a microplate reader SpectraFluor Plus (TECAN, Mannedorf, Switzerland). Readings were done in quadruplicates. Three independent experiments were performed for each measurement. Normalized fluorescence data are presented as means ± SEM.

Additionally, we evaluated the lysosomal integrity microscopically. Cells were seeded in 6-channel µ-Slides (Ibidi, Martinsried) at density 15,000 cells per well. Then cells were stimulated with NPs for 24 h. After, cells were labeled with 5 µg mL^−1^ AO in culture medium for 15 min at 37 °C and imaged using spinning disk confocal microscopy IXplore SpinSR (Olympus, Tokyo, Japan). As positive control treatment with 20 % ethanol for 10 min was used.

### 2.10. Assessment of Mitochondrial Membrane Potential

Cells were seeded in 6-channel µ-Slides (Ibidi, Martinsried) at density 15,000 cells per well. Then cells were treated with different NP concentrations for indicated periods of time. Afterwards, cells were stained with 1 µM JC-1 probe, and imaged using spinning disk confocal microscopy IXplore SpinSR (Olympus, Tokyo, Japan). As positive control, treatment with 20% ethanol for 20 min was used. JC-1 is very selective and sensitive dye to assess mitochondria potential, which enters into mitochondria and reversibly changes color from red to green, as the membrane potential decreases [43]. When cells have high mitochondrial ΔmΦ, JC-1 spontaneously forms complexes known as J-aggregates with intense red fluorescence. On the other hand, in cells with low ΔmΦ, JC-1 remains in the monomeric form, which shows mostly green fluorescence.

### 2.11. Cell Extracts and Western Blot Analysis

Aliquots of whole cell lysates containing equal amounts of protein were obtained using lysis buffer RIPA (Millipore, Burlington, MA, USA) in accordance with the manufacturer’s instructions and our verified protocol [14,44]. For isolation of nuclear and cytoplasmic extracts from the cells NE-PER Nuclear and Cytoplasmic Extraction Kit (Thermo Fisher Scientific) was used according to the manufacturer’s guidelines. Protein lysates were subjected to SDS-PAGE electrophoresis, and transferred to PVDF membranes. The membranes were blocked with 5% (*w*/*v*) fat free dried milk or alternatively with 5% (*w*/*v*) bovine serum albumin (BSA) for 1 h. Afterwards, membranes were incubated with various specific primary antibodies listed in Supplementary Table S2 at 4 °C overnight. Chemiluminescence signals were detected using an imaging system GBOX CHEMI XRQ (Syngene, Synoptics group, Cambridge, UK) and acquisition software GeneTools (Syngene, Synoptics group). Densitometric quantification of blots was done using GeneTools quantification software (Syngene, Synoptics group).

### 2.12. Detection of Intracellular ROS Levels

ROS levels were measured using Cellular ROS/Superoxide Detection Assay Kit (Abcam, Cambridge, United Kingdom). Briefly, cells were seeded in 6-channel µ-Slides (Ibidi, Martinsried) at density 15,000 cells per well and treated with 50 μg Fe mL^−1^ NPs for 24 h. Following this, cells were labeled with Oxidative Stress Detection Reagent (Green) for ROS detection and Superoxide Detection Reagent (Orange) according to the manufacturer’s instructions (Abcam, Cambridge, United Kingdom). Stained cells were imaged using spinning disk confocal microscopy IXplore SpinSR (Olympus, Tokyo, Japan). As positive control treatment with 1 mM H_2_O_2_ for 30 min was used.

### 2.13. Nanoparticle Quantifications from Confocal Microscopy Images

For intracellular nanoparticle quantification, cells were seeded in 6-channel µ-Slides (Ibidi, Martinsried) and incubated with different concentrations of fluorescently-labelled NPs. Then, cells were stained with CellMask Orange (Thermo Fisher Scientific) and Hoechst 33342 (Thermo Fisher Scientific). Stained cells were imaged using spinning disk confocal microscopy IXplore SpinSR (Olympus, Tokyo, Japan). The stacks of confocal cross-sections obtained by confocal microscopy were evaluated applying the digital method Particle_in_Cell-3D based on the ImageJ software and free to download at ImageJ Documentation Portal [45]. By applying this method, intra- and extracellular space were automatically differentiated. The confocal fluorescence image of the cell membrane is transformed into a mask of the cell in each measured confocal plane. By applying this mask to the corresponding particle image, the NPs are classified. The method automatically differentiates between NPs in the intracellular and extracellular space and NPs close to the cellular membrane, the transition or enlarged membrane region. NPs which are present in this transition region are passing the first step of cellular uptake. As NPs are often present as clusters or aggregates inside the cell, it is not possible to quantify them by counting the individual spots. This problem is solved by using the fluorescence intensities to estimate the number of NPs. It is then possible to estimate the absolute number of NPs that has been taken up into the cytoplasm [46].

### 2.14. Super-Resolution Spinning Disk Confocal Microscopy

In order to perform super-resolution imaging of cytoskeleton and lysosomes, we utilized novel IXplore SpinSR Olympus super-resolution imaging system (Olympus, Tokyo, Japan). Cells were seeded in 6-channel µ-Slides (Ibidi, Martinsried) and incubated with different concentrations of fluorescently-labelled NPs. Then cells were stained for F-actin, tubulin or LysoTracker™ Red DND-99. Dilutions and catalogue numbers of used antibodies and chemicals are given in Supplementary Tables S1 and S2. Fluorescence images were taken with the acquisition software cellSens (Olympus, Tokyo, Japan). ImageJ software (NIH) was used for image processing and quantification.

### 2.15. Statistical Analysis

Quantitative results are present as mean ± SEM. The statistical significance of differences between the groups was determined using ANOVA with subsequent application of Newman-Keuls test. All statistical analyses were performed using MaxStat Pro 3.6. Differences were considered statistically significant at (*)*p* < 0.05.

Fluorescence microscopy analysis (namely analysis of lysosomal size and circularity, colocalization of proteins Rab7/LAMP1, cellular localization of p53) was subjected to quantitative assessment in accordance with rigorously defined guidelines [47]. For a quantitative analysis of the images, we utilized the published guidance for quantitative confocal microscopy [48,49]. Images from three independent experiments were subjected to quantitative analysis. In each experiment 10 randomly selected fields from each sample were imaged. In order to determine sample size, we utilized a previously described statistical method [50]. According to this method, the sample size for 95 % confidence level and 0.8 statistical power corresponds to 20. Thus, at least 20 randomly selected cells were used in fluorescence microscopy quantification.

The sample size determination was assessed utilizing a statistical method described in [50], taking into assumption 95% confidence level and 0.9 statistical power.

## 3. Results

### 3.1. Effect of IRON Oxide Nanoparticles on Cell Viability and Oxidative Stress

As a model of NPs, we selected previously well-characterized core-shell iron oxide nanoparticles coated with carboxymethyldextran shell (mean hydrodynamic diameter of about 200 nm) [7,30,31,32,33]. This selection was done due to physiological relevance of such type of NPs. Indeed, iron oxide NPs with dextran-based shell with diameter larger than 200 nm are known to be rapidly (a plasma half-life of less than 10 min) accumulate in the liver [1,51,52]. This makes such particles an attractive candidate as MRI contrast agent for liver imaging [1,51,52,53]. In fact, Kupffer cells have been shown to take up NPs on a broad size scale as first line of uptake [14,19,53,54]. However, recent studies indicate that particles with relatively big diameter comparable with liver sinusoidal fenestrations (~150–200 nm) can penetrate the space of Disse and directly interact with hepatocytes [19,20]. Surprisingly, in literature there are very few reports about responses of hepatic cells to sub-lethal treatment with NPs, for review see [19]. Moreover, most of the research is done utilizing only one cell line without direct comparison of the observed effects on closely related cell lines [16,19,20]. Therefore, in this study, we chose three hepatic cell lines (HepG2, Huh7, and Alexander cells).

The physicochemical properties of the nanoparticles investigated in this study are summarized in Figure S1. The physicochemical analysis revealed that both the fluorescent and unlabeled NPs have a comparable hydrodynamic diameter around 200 nm (Supplementary Figure S1b,c), which was doubled for both particles after 2 h incubation in medium with 10% serum (Supplementary Figure S1b,c). Fluorescent and unlabeled NPs had a slightly negative zeta potential ~ ‒2 mV (Supplementary Figure S1c). After incubation with the medium both NPs showed similar zeta potential change (Supplementary Figure S1c). Thus, these data imply that NP labeling had no impact on size and zeta potential of the NPs. Of note, it is well known that in protein-rich liquids NPs become coated with proteins and other biomolecules, which results in formation of so-called protein corona [55]. Protein corona may play an important role in determining subsequent cellular responses to NP treatment [55], including effects on mTOR signaling [56]. However, the used NPs showed very weak zeta potential (Supplementary Figure S1c). Such potential resulted in fast protein corona formation that was independent of NP concentration (Supplementary Figure S2).

**Figure 1:**
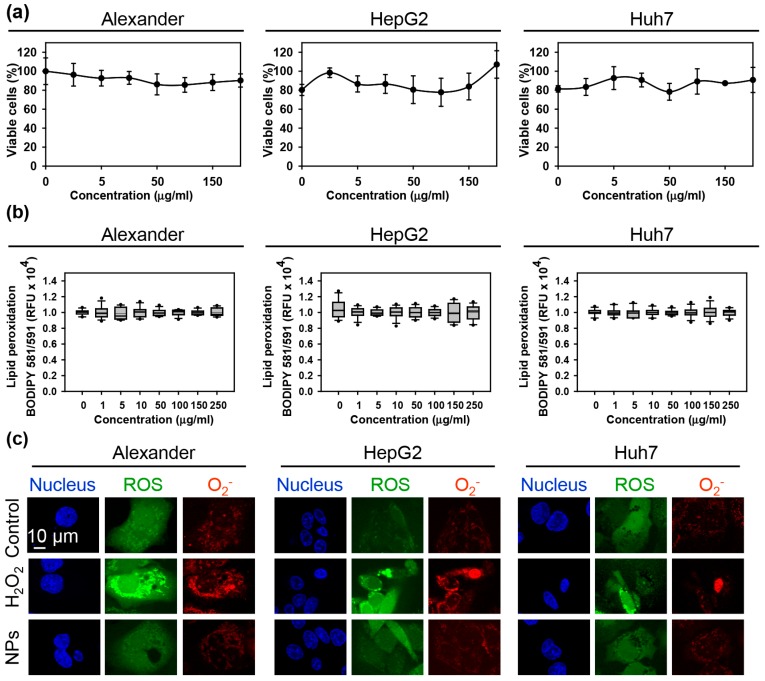
Assessment of the nanoparticle uptake and biocompatibility in three distinct hepatic cell lines. (**a**) Cytotoxicity of nanoparticles in three distinct cell lines: Alexander, HepG2, Huh7. Cytotoxicity was assessed using alamarBlue assay. Data are expressed as means ± SEM (*n* = 4). (**b**) Detection of lipid peroxidation using BODIPY™ 581/591 C11 lipid peroxidation sensor (ThermoFisher Scientific). Data are expressed as means ± SEM (*n* = 3). (**c**) NP treatment did not induce intracellular ROS/Superoxide (O_2_^−^) production and different subcellular accumulation. Cells were treated for 24 h with nanoparticles 50 μg Fe mL^−1^. NP-treated cells were stained with ROS/Superoxide Detection Assay Kit and imaged by confocal microscopy. Representative images out of three independent experiments are shown. Positive control 1 mM H_2_O_2_ for 30 min was used.

First, we confirmed that the sub-lethal treatment of three cell lines cells with the NPs had no toxic response during 24 h treatment (Figure 1a). Moreover, there was no observable oxidative stress upon the treatment with NPs (Figure 1b). Additionally, we analyzed the accumulation of intracellular ROS followed by NP treatment. We used distinct fluorescent probes for total ROS and superoxide anion (O_2_^−^). Indeed, neither total ROS nor superoxide were elevated upon NP treatment (Figure 1c and Supplementary Figure S3). Contrarily, positive control (treatment with 1 mM H_2_O_2_) treatment showed marked elevation of total ROS and superoxide in all three cell lines (Figure 1c and Supplementary Figure S3). These data confirmed the absence of oxidative stress upon the treatment with NPs.

**Figure 2:**
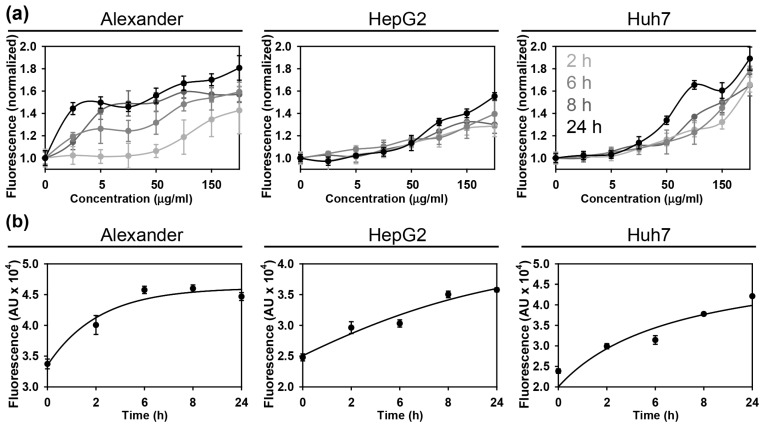
Assessment of the nanoparticle uptake in three distinct hepatic cell lines. Assessment of the uptake kinetics of fluorescently labeled nanoparticles by Huh7, HepG2 and Alexander cells. The fluorescence intensity increase upon nanoparticle treatment was detected by a fluorescent microplate reader. (**a**) depicts concentration-dependent uptake kinetics. (**b**) shows time-dependent uptake kinetics, when cells were treated with NPs 100 μg Fe mL^−1^ for indicated periods of time. The data are expressed as mean  ±  SEM, *n* = 4 each.

Next, we checked the uptake kinetics of the NP to be sure that within given time frame cell take enough material. Indeed, all three cell lines showed concentration- and time-dependent uptake of NPs (Figure 2a,b). We observed uptake saturation after 12 h of incubation with 100 μg Fe mL^−1^ of NPs (Figure 2b). In fact, after the intravenous injection of physiologically relevant doses of iron oxide nanoparticles (10–40 mmol Fe/kg), blood levels may reach up to 50 μg Fe mL^−1^ and within 30 min after injection nanoparticles are taken up by liver [14,57,58,59].

### 3.2. Nanoparticle Uptake and Cytoskeleton Remodeling

**Figure 3:**
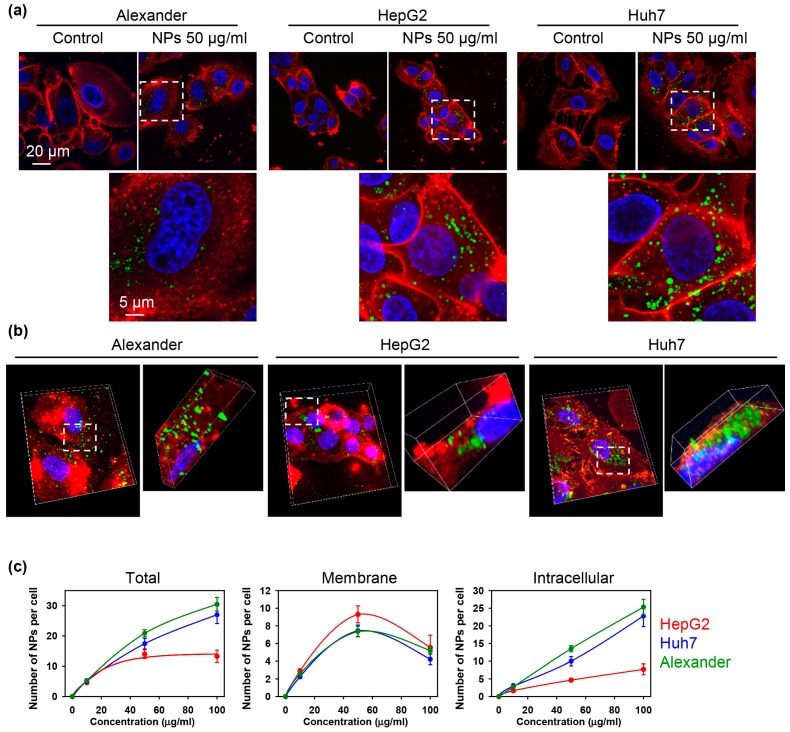
Quantification of the nanoparticle uptake in three distinct hepatic cell lines. (**a**) Representative high-resolution confocal images of cells incubated with nanoparticles. Huh7, HepG2 and Alexander cells were treated for 12 h with NPs 50 μg Fe mL^−1^ (green). Cell membranes were labeled with CellMask™ Orange (red). (**b**) 3D reconstruction of NP-labelled cells. Cells were treated like in (**a**) and 3D rendering was done using ImageJ software. (**c**) Quantification of nanoparticle uptake. Cells were treated with different nanoparticle concentrations for 12 h. After the treatment cells were labeled with CellMask™ Orange and imaged using IXplore SpinSR Olympus microscopy system. Number of particles per cell was calculated using image processing by Particle_in_Cell_3D macro in ImageJ. Data are expressed as mean ± SEM of at least three independent experiments (*n* = 30 cells).

Further, to confirm sub-cellular localization of NPs after uptake we performed high-resolution confocal microscopy (Figure 3a). 3D reconstruction of the optical sectioning confirmed that NPs were distributed inside cellular volume already after 12 h of treatment (Figure 3b).

Additionally, we quantified NP number adsorbed to the membrane and ingested by the cells (Figure 3c). This quantification confirmed dose-dependent nanoparticle uptake in all three cell lines (Figure 3c). Indeed, HepG2 showed less capability for NP internalization comparing with Alexander and Huh7 cells (Figure 3c). It is worth noting here that we did not find any observable changes in overall shape or size of the cells upon NP uptake in comparison with control (Figure 3a).

**Figure 4:**
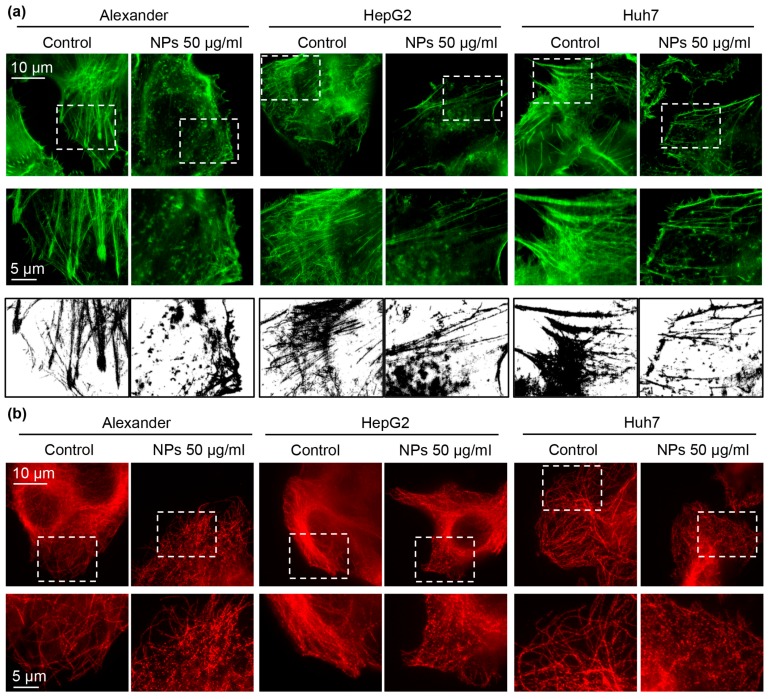
Cytoskeleton remodeling under NP treatment. (**a**) IXplore SpinSR Olympus super-resolution microscopy of F-actin staining (green). Cells were treated for 24 h with nanoparticles 50 μg Fe mL^−1^, fixed and stained for F-actin. Binarization was done using ImageJ software. (**b**) Cells were treated with NPs as in a. After the treatment cells were fixed and stained for tubulin (red). Super-resolution imaging was done using IXplore SpinSR Olympus super-resolution system.

Recent studies have shown that NPs may induce cytoskeletal remodeling without oxidative stress involvement [17,60,61]. Thus, we checked whether treatment with NPs for 24 h results in cytoskeletal changes. Indeed, already conventional confocal microscopy revealed substantial re-organization of F-actin and tubulin (Supplementary Figure S4). To clearly see the cytoskeletal remodeling under higher resolution, we utilized novel super-resolution spinning disk microscopy. This type of microscopy is quite similar to structured illumination microscopy and reaches a spatial resolution of 120 nm [62]. Super-resolution imaging confirmed F-actin and tubulin remodeling under NP treatment in all three cell lines (Figure 4a,b).

### 3.3. Nanoparticles Alter Lysosomal Function

It is widely accepted that cells engulf nanomaterials by endocytosis process and finally traffic them to the lysosomes [63,64,65]. Thus, it is not a surprise that lysosomes play a crucial role in determining the fate of the nanomaterial [63,64,65]. Overall, lysosomes have been found to actively participate and regulate important parts of cellular metabolism, such as nutrient sensing, transcriptional regulation, and metabolic homeostasis [27,66,67].

Recent studies even postulate the lysosome-centric signaling networks as pivotal hub for nutrient sensing and metabolic adaptation, for review see [27]. Taking all these together, we hypothesized that lysosomes are a central hub for iron oxide nanoparticle-mediated cellular signaling that is tightly dependent on the genetic background of the affected cells. Firstly, we assessed that NPs end up in lysosomes.

**Figure 5:**
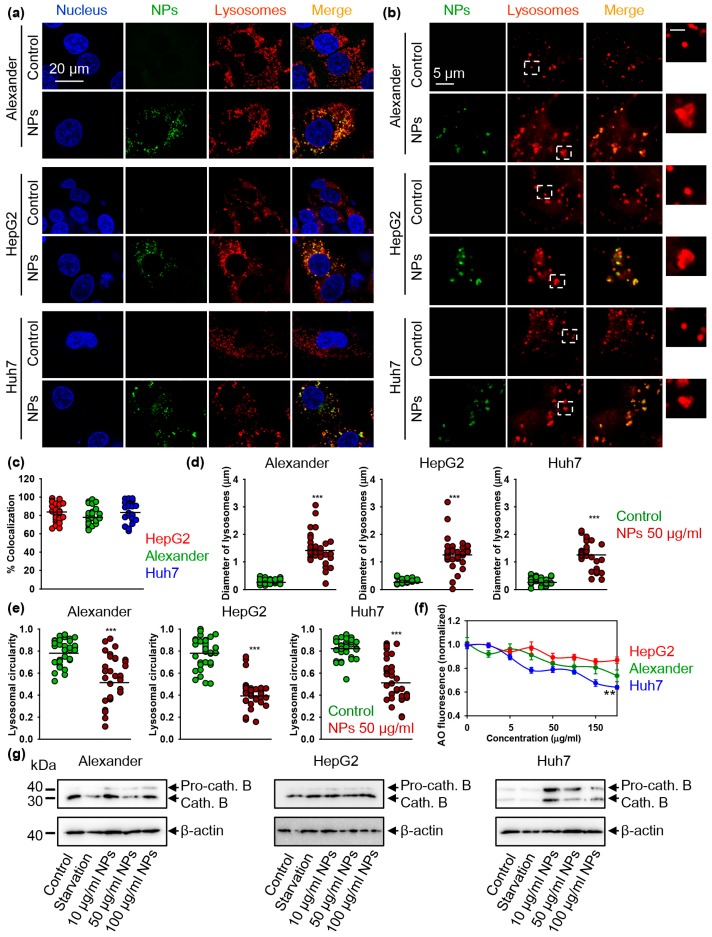
Lysosomal impairment induced by nanoparticles. (**a**) Localization of fluorescently labeled nanoparticles (green) in lysosomal compartments. Cells were treated for 12 h with nanoparticles 50 μg Fe mL^−1^ and labeled with LysoTracker™ Red DND-99 (red). Nuclei were stained with hoechst 33342 nuclear stain (blue). Merge of green and red gives yellow color. Labeled cells were then imaged using spinning disk confocal microscopy. (**b**) IXplore SpinSR Olympus super-resolution microscopy of lysosomes. Cells were treated for 12 h with nanoparticles 50 μg Fe mL^−1^ (green) and labeled with LysoTracker™ Red DND-99 (red). Scale bar for the zoomed insert is 1 µm. (**c**) Colocalization analysis of nanoparticles and lysosomes from images (**a**) is presented. Quantifications performed using ImageJ are presented as means of *n* = 25 cells. (**d**) Measurements of the lysosomal diameter upon nanoparticle uptake. Super-resolution images (**b**) were quantified using ImageJ software. Quantifications are presented as means of *n* = 34 cells. (***) *p* < 0.001 denotes significant differences with respect to control (no particles treatment). (**e**) Assessment of lysosomal circularity out of super-resolution images (**b**). Circularity was calculated using ImageJ software. Quantifications are presented as means of *n* = 34 cells. (***) *p* < 0.001 denotes significant differences with respect to control (no particles treatment). (**f**) Lysosomal integrity as measured by acridine orange (AO) red fluorescence decrease. Cells were treated with indicated concentrations of nanoparticles for 24 h, stained with AO and then the fluorescence intensity was measured using a fluorescent microplate reader. The data are expressed as mean ± SEM, *n* = 3 each. (**) *p* < 0.01 denotes significant differences. (**g**) Nanoparticles impair maturation of pro-cathepsin B in Alexander and Huh7 cells but not in HepG2. Cells were stimulated with nanoparticles at indicated concentrations for 12 h. Expression of cathepsin B was analyzed by immunoblotting. Positive control—serum starvation for 12 h. Actin denotes loading control. Densitometric quantification of blots is shown in Figure S7 of Supplemental Materials.

Confocal microscopy study revealed lysosomal localization of NPs after the treatment (Figure 5a,c and Supplementary Figure S5). Moreover, accumulation of NPs in lysosomes led to lysosomal size and shape alterations (Figure 5b,d,e). Upon NP treatment, lysosomes in all three cell lines increased their size significantly (Figure 5b,d). Additionally, the circular shape of lysosomes under NP treatment changed to more irregular one (Figure 5b,e). Importantly, those alterations in lysosomal size and shape were very similar in all three cell lines. However, when we checked lysosomal acidification utilizing the acridine orange lysosomal integrity assay, lysosomal activity was significantly impaired in Alexander and Huh7 cells (Figure 5f). Indeed, lysosomal acidification impairment caused by NPs was most profound in Huh7 cells (Figure 5f). Importantly, the observed NP-impaired lysosomal activity was mild and did not result in strong lysosomal damage as compared to positive control treatment (Supplementary Figure S6). In fact, it is well known that strong lysosomal damage decreases of the autophagic flux [68,69,70], whereas mild lysosomal impairment promotes autophagy as a surviving mechanism [71]. This is exactly what we observed in Huh7 cells upon NP treatment.

A range of biological functions is regulated by lysosomal cathepsins. Deregulation of cathepsins (specifically, cathepsins B) is a validated hallmark of lysosomal dysfunction [72,73]. Therefore, we analyzed how NP treatment affect cathepsins B. Consistent with lysosomal integrity (Figure 5f and Supplementary Figure S6), there was no noticeable effect on cathepsins B expression in HepG2 cells (Figure 5g and Supplementary Figure S7). However, Alexander and Huh7 cells treated with NPs showed increased expression of cathepsins B (Figure 5g and Supplementary Figure S7). Moreover, in both cell lines NPs inhibited conversion of pro-cathepsin B into the proteolytically active mature form (Figure 5g and Supplementary Figure S7). These data clearly indicate that NP treatment results in lysosomal dysfunction in Alexander and Huh7 cells. In line with lysosomal integrity observations (Figure 5f and Supplementary Figure S6), Huh7 were the most sensitive in terms of lysosomal dysfunction upon NP treatment (Figure 5g and Supplementary Figure S7). As a result of severe lysosomal damage, cathepsins are released from lysosomal lumen to cytosol and cause damage of mitochondria, which augment autophagy [68,69,70]. Thus, we checked mitochondria damage by JC-1 staining. Indeed, NP treatment resulted in reduction of mitochondrial membrane potential (ΔmΦ) in Alexander and Huh7 cells, as evidenced by the fluorescent intensity decrease of retained JC-1 red aggregates (Supplementary Figure S8). However, we did not observe any changes in the mitochondrial integrity in response to NPs (Supplementary Figure S8). Thus, these data confirm only mild lysosomal impairment induced by NPs.

**Figure 6:**
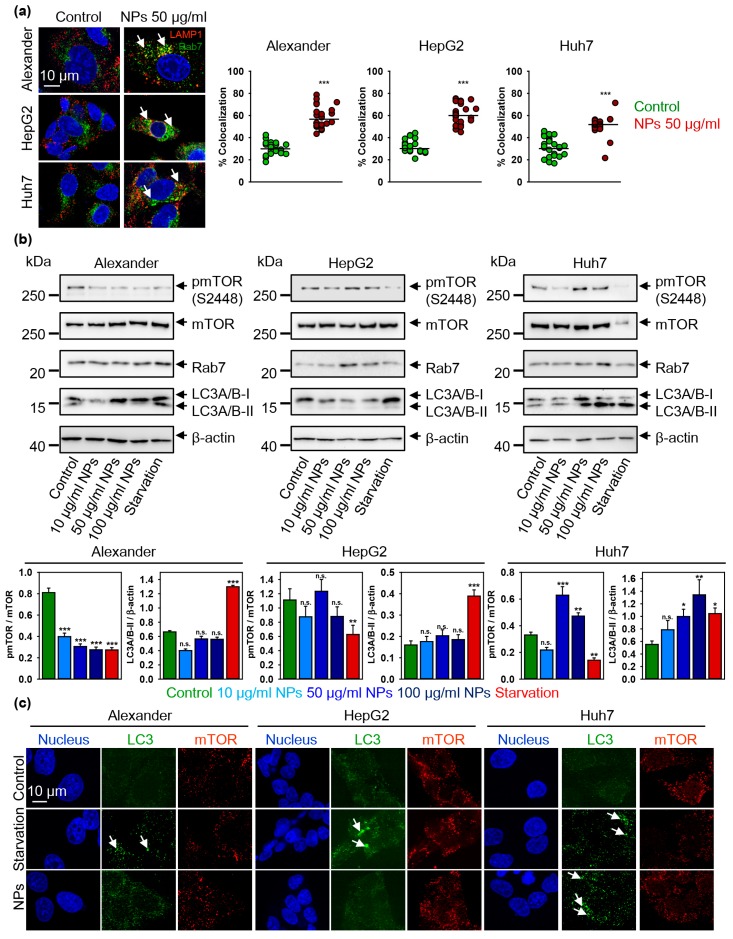
Nanoparticle treatment-induced autophagic flux is cell line dependent. (**a**) Colocalization analysis of nanoparticles and Rab7 protein. Cells were treated for 12 h with nanoparticles 50 μg Fe mL^−1^, fixed and immunostained for LAMP1 (red) and Rab7 (green). Labeled cells were then imaged using spinning disk confocal microscopy. Colocalization quantifications were done in ImageJ and presented as means of *n* = 25 cells. (***) *p* < 0.001 denotes significant differences with respect to control (no particles treatment). (**b**) Cells were stimulated with nanoparticles at indicated concentrations for 12 h. Expressions of pmTOR, mTOR, Rab7 and LC-3 were analyzed by immunoblotting. Positive control—serum starvation for 12 (Alexander, Huh7) and 14 (HepG2) h. Actin denotes loading control. Graphs show densitometric quantification of blots. (*) *p* < 0.05, (**) *p* < 0.01 and (***) *p* < 0.001 denotes significant differences with respect to control (no particles treatment). (**c**) Confirmation of autophagic flux by formation of cellular autophagosome punctae containing LC3-II. Cells were treated for 12 h with nanoparticles 50 μg Fe mL^−1^, fixed and immunostained for mTOR (red) and LC3 (green). Labeled cells were then imaged using spinning disk confocal microscopy. Arrows indicate formation of cellular autophagosome punctae. Positive control—serum starvation for 12 (Alexander, Huh7) and 14 (HepG2) h. Nuclei were stained with Hoechst 33342.

The end point of NP trafficking by the cell is a formation of mature endolysosomes [63,64,65], which are characterized by the presence of Rab7/LAMP1-vesicles [74,75]. Indeed, NPs treatment lead to significant increase in Rab7/LAMP1 colocalization in all three cell lines (Figure 6a and Supplementary Figure S9), indicating that NP treatment leads to the formation of terminal endocytic vesicles. Interestingly, in HepG2 and Huh7 cells NP treatment resulted in increased expression of total Rab7 protein (Figure 6b), whereas Alexander cell showed no differences in Rab7 expression (Figure 6b).

### 3.4. Iron Oxide Nanoparticles Modulate Autophagic Flux and Mtor Activity in Hepatic Cells

Emerging evidence suggest that various types of nanoparticles, including iron oxide, modulate autophagy in a lysosome-dependent manner [28,63,71,76,77,78]. Indeed, it is widely accepted that NPs may induce autophagy dysfunction via either overstimulation of autophagic flux or inhibition of autophagosome degradation [28,63,71,76,77,78]. In fact, the detailed mechanism of how iron oxide NPs affect autophagy function is not fully understood. Therefore, we further evaluated how iron oxide NP affect the activity of crucial autophagy-related proteins. Expression of LC3 protein represents a classical autophagic marker [79,80].

Indeed, consistently, with the increased cathepsins B expression (Figure 5g) and mild lysosomal impairment (Figure 5f and Supplementary Figure S6) we found elevated levels of LC3 protein upon NP treatment in Alexander and Huh7 cells (Figure 6b). In line with autophagic flux induction in Alexander cells, we found down-regulation of mTOR phosphorylation (Figure 6b).

In fact, the mammalian target of the rapamycin (mTOR) is a well-known regulator of autophagy, which elevated enzymatic activity in phosphorylated form inhibits autophagy [26,27]. When autophagic flux is executed, LC3–phosphatidylethanolamine conjugate (LC3-II) accumulates in autophagosome membranes, which can be detected by the puncta formation in microscopy immunofluorescent analysis [79,80]. Indeed, in Alexander cells NP treatment did not result in lipidation of LC3 (Figure 6b,c and Supplementary Figure S10). Contrary, NP treatment in Huh7 cells resulted in autophagic flux execution confirmed by the accumulation of lipid-conjugated LC3 (Figure 6b,c and Supplementary Figure S10). In order to validate autophagic flux in Huh7, we utilized inhibitor of lysosomal degradation (bafilomycin A_1_). Indeed, co-treatment of Huh7 with NPs and bafilomycin A_1_ increased the number of LC3-positive puncta confirming activated autophagy (Supplementary Figure S11). These data clearly indicate distinct autophagic responses in hepatic cells. In Alexander cells one can see deregulation of autophagy upon NP treatment, whereas in Huh7 nanoparticles support autophagic flux. However, unexpectedly, NPs induced elevated mTOR phosphorylation in Huh7 cells (Figure 6b).

It is worth noting here, that HepG2 cells showed neither significant increase in LC3 protein levels, nor LC3 lipidation (Figure 6b,c and Supplementary Figure S10). HepG2 were as well insensitive to NP treatment in terms of mTOR phosphorylation (Figure 6b).

**Figure 7:**
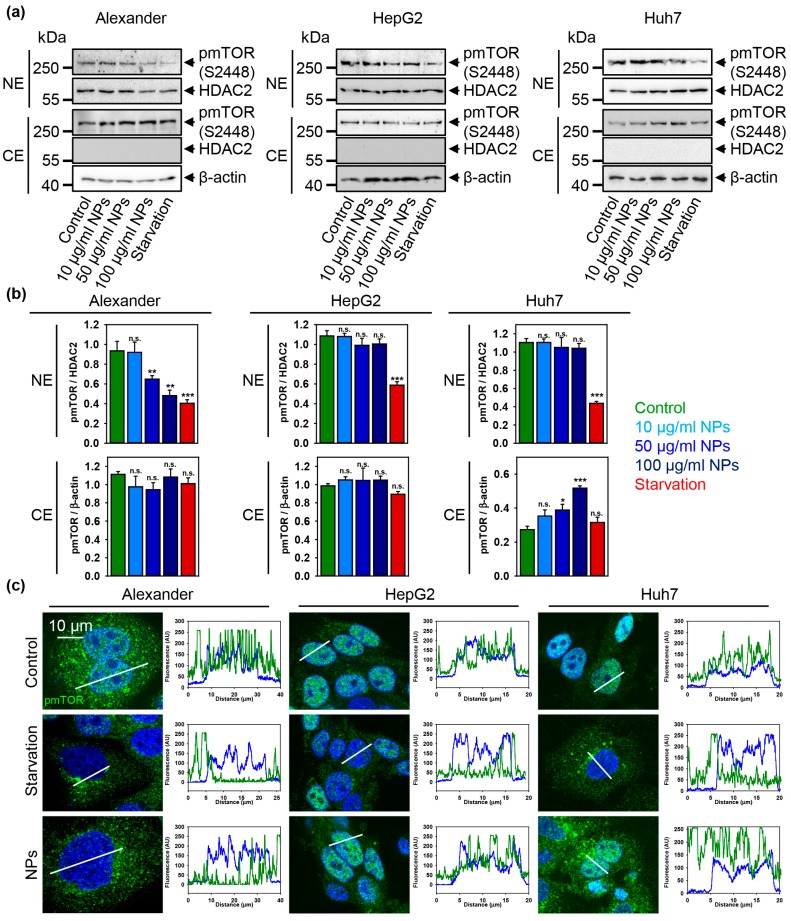
Sub-cellular localization of pmTOR is affected by nanoparticles. (**a**) The cytosolic (CE) and nuclear extracts (NE) from cells treated with indicated concentrations of nanoparticles. Cells were treated for 12 h with nanoparticles. HDAC2 serves as a nuclear marker. Expressions of pmTOR was analyzed by immunoblotting. Positive control—serum starvation for 12 (Alexander, Huh7) and 14 (HepG2) h. Actin denotes loading control. (**b**) Densitometric quantification of blots (**a**). The data are expressed as mean ± SEM. (*) *p* < 0.05, (**) *p* < 0.01 and (***) *p* < 0.001 denotes significant differences with respect to control (no particles treatment). (**c**) Sub-cellular localization of pmTOR upon nanoparticle treatment. Representative confocal microscopic images and linescans of three cell lines. Cells were treated for 12 h with nanoparticles 50 μg Fe mL^−1^, fixed and immunostained for pmTOR (green). Positive control—serum starvation for 12 (Alexander, Huh7) and 14 (HepG2) h. Nuclei were stained with Hoechst 33342.

A number of studies clearly indicate that NPs modulate mTOR activity [18,81,82]. Still, the precise molecular mechanisms of mTOR signaling affected by NPs are largely unknown [18]. Therefore, we further analyzed how sub-cellular distribution and activity of mTOR might be altered by NPs. Consistently with all above discussed data, we found no changes in mTOR activity neither in the nucleus nor in the cytosol of HepG2 cells treated with NPs (Figure 7a–c and Supplementary Figure S12). Interestingly, in Alexander cells NP treatment resulted in down-regulation of mTOR phosphorylation in the nucleus but not in cytosol (Figure 7a–c and Supplementary Figure S12). Contrary, Huh7 treatment with NPs led to increased mTOR activity in cytosol, whereas nuclear mTOR levels were un-changed (Figure 7a–c and Supplementary Figure S12). As a matter of fact, classical mTOR signaling is determined at the lysosomes, where mTORC1 is phosphorylated at the lysosomal surface [25,26,27]. Of note, a number of studies showed that phosphorylated mTOR can be found in nucleus [83,84,85,86,87,88], but the exact role of mTOR in the nucleus remains enigmatic [84,87]. Indeed, increased mTOR nuclear localization has been associated with poor prognosis in patients with different types of cancer [86,89].

### 3.5. p53 Sub-Cellular Localization Mediates Hepatic Cell Response to IRON Oxide Nanoparticles

The fact, that NP-cell interactions vary greatly in distinct cell types, is widely accepted [16,63,64,65,90,91]. However, studies that directly compare NP-mediated effects in different cell lines of the same lineage are still rather limited. Summarizing all the above discussed results, we can clearly conclude that iron oxide NPs trigger very distinct, however related, signaling events in three hepatic cell lines. Therefore, the next logical step is to evaluate what the molecular prerequisites of such different responses are.

It is worth noting here, that Huh7 cells express the highest level of p53 protein in comparison with Alexander and HepG2 cells [34,92,93,94]. Therefore, we checked the sub-cellular distribution of p53 protein in Alexander, HepG2, and Huh7 cells.

**Figure 8:**
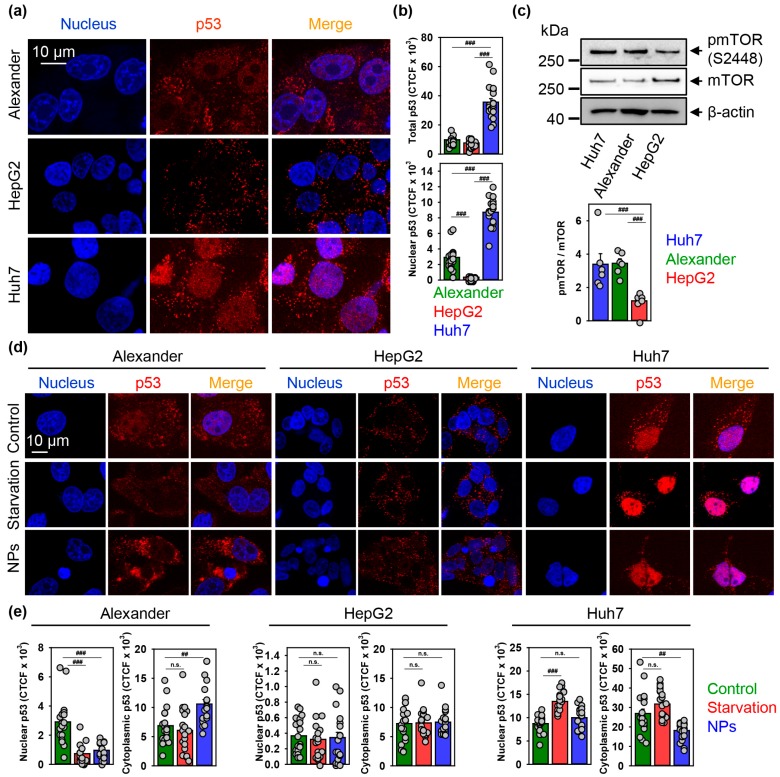
Nanoparticles affect p53 nuclear shuttling. (**a**,**b**) Representative confocal microscopic images and quantification of p53 sub-cellular localization in distinct cell lines. Huh7, HepG2 and Alexander cells were fixed and immunostained for p53 (red). Nuclei were stained with Hoechst 33342. Labeled cells were then imaged using spinning disk confocal microscopy (**a**). Quantification of p53 cellular fluorescence (**b**) was done by ImageJ and presented as means of *n* = 20 cells. (###) *p* < 0.001 denotes significant differences. (**c**) Expressions of pmTOR and mTOR were analyzed in whole cell lysates of HepG2, Huh7, Alexander cells by immunoblotting. Actin—control of equal protein loading. The graphs show densitometric quantification of pmTOR/mTOR ratio. Quantification performed using ImageJ. The data are expressed as mean ± SEM, *n* = 5–6. (###) *p* < 0.001 denotes significant differences. (**d**,**e**) Representative confocal microscopic images and quantification of p53 sub-cellular localization in distinct cell lines upon nanoparticle treatment. Cells were treated for 12 h with nanoparticles 50 μg Fe mL^−1^, fixed and immunostained for p53 (red). Labeled cells were then imaged using spinning disk confocal microscopy (**d**). Positive control—serum starvation for 12 h. Nuclei were stained with Hoechst 33342. Quantification of p53 cellular fluorescence (**e**) was done by ImageJ and presented as means of *n* = 20 cells. (##) *p* < 0.01 and (###) *p* < 0.001 denote significant differences.

Quantitative confocal imaging analysis revealed that Huh7 express the highest total p53 protein levels (Figure 8a,b and Supplementary Figure S13). Additionally, Alexander and Huh7 cells showed nuclear localization of p53, whereas HepG2 had only cytosolic p53 (Figure 8a,b). Again, nuclear p53 protein levels were significantly higher in Huh7 in comparison with Alexander cells (Figure 8a,b).

Of note, both Huh7 and Alexander cell express mutated p53, and in contrast, HepG2 cells have wild type form of p53 [92,93]. In addition, mutated p53 has been shown to enhance the mTOR activity [95,96]. Thus, we compared level of mTOR endogenous phosphorylation in three cell lines. Indeed, in cells with mutated p53, namely Alexander and Huh7, ratio of phosphorylated TOR protein to total mTOR level was significantly higher than in HepG2 (Figure 8c). In contrast, HepG2 cells show neither elevated mTOR endogenous phosphorylation (Figure 8c) nor nuclear p53 localization (Figure 8a,b) comparing to Huh7 and Alexander cells. However, HepG2 cells possess elevated levels of the anti-apoptotic Bcl-2 comparing to Huh7 and Alexander cells [34,94,97]. Taking into account the reported fluctuations concerning Bcl-2 expression in HepG2 cells in the literature [98,99], we analyzed Bcl-2 protein levels in all three cell lines. Immunoblot analysis of Bcl-2 expression revealed, that HepG2 express higher levels of the anti-apoptotic Bcl-2 when compared to Huh7 and Alexander cells (Supplementary Figure S14).

In fact, Bcl-2 shows as well anti-autophagic activity by stabilizing lysosomal membrane and preventing lysosomal destabilization [100,101]. As a result, cancer cells, that express elevated Bcl-2 levels, are more chemoresistant to the standard treatments that activate either apoptosis or autophagic cell death [102,103]. Not surprisingly, we found that HepG2 cells upon NP treatment did not exhibit lysosomal dysfunction (Figure 5g) and destabilization (Figure 5f and Supplementary Figure S6). Additionally, cytoskeletal reorganization upon NP treatment in HepG2 is mild (Figure 4). In fact, cytoskeletal dynamics is crucial in autophagy progression [104]. Thus, mild cytoskeletal reorganization attenuates autophagy execution. It was shown that Bcl-2 family members may affect cytoskeletal dynamics and cell movement [105]. Therefore, it may be plausible that high Bcl-2 levels influence cytoskeletal reorganization in HepG2 cells. However, NP treatment resulted in formation of terminal endocytic vesicles (Figure 6a), this in turn did not lead to execution of autophagy (Figure 6b,c).

Contrary to HepG2, endogenously elevated mTOR phosphorylation (Figure 8c) together with p53 mutation in Alexander and Huh7 cells make those cell lines susceptible to lysosomal dysfunction triggered by iron oxide NPs. However, Alexander cells in response to NPs showed impaired autophagy execution comparing with Huh7 (Figure 6b,c). Taking into account active role of p53 in autophagy regulation and crosstalk with mTOR signaling axis [95,96,106,107,108], we analyzed how NP treatment would affect p53 sub-cellular localization. Indeed, NPs decreased nuclear and increased cytosolic localization of p53 in Alexander cells (Figure 8d,e and Supplementary Figure S15). Treatment of Huh7 cells led to significant decrease of cytosolic p53 (Figure 8d,e and Supplementary Figure S15). Consistent with data on autophagy activation (Figure 6b,c) and lysosomal dysfunction (Figure 5f,g and Supplementary Figure S6), HepG2 cells were nonresponsive in terms of p53 localization under NP treatment (Figure 8d,e and Supplementary Figure S15).

## 4. Discussion

Iron oxide NPs with a dextran-based shell are known to elicit delayed cytotoxic effects in various cell types via excessive accumulation of reactive oxygen species [12,13,14,15,58,109,110,111,112,113]. However, recent studies indicate that sub-lethal treatment of cells with different types of NPs leads to observable morphological changes [17,60,61,114,115]. In addition, even nanoparticle concentrations that do not induce oxidative stress alter cellular morphology significantly [17]. However, the origin of a signal that drives morphological changes upon sub-lethal treatment of cells with NPs remains unclear.

Summarizing all the data, we can derive that sub-lethal treatment with iron oxide NP leads to NP accumulation in lysosomes. This lysosomal accumulation of NPs induces alterations in lysosomal size and shape and progressive impairment of lysosomal function. Such lysosomal impairment is, in fact, mild comparing to dramatic progressive LMP caused by ethanol treatment (Supplementary Figure S6). Mild lysosomal dysfunction has been shown to support autophagic flux [71], which is in line with our findings. The resulting lysosomal dysfunction affects sub-cellular localization of pmTOR and p53. Progressive lysosomal dysfunction leads to initiation of autophagic flux, which is supported by the nuclear p53. In contrast, cytosolic p53 and high levels of Bcl-2 inhibit autophagy.

Of note, Alexander cells upon NP treatment show elevation of cathepsin B (Figure 5g) and LC3 levels (Figure 6b), but do not bear lipidated form of LC3 (Figure 6c). Importantly, NP treatment of Alexander cells leads to contemporary high cytoplasmic levels of p53 (Figure 8d,e). It should be stressed here that the subcellular localization of p53 differently affects autophagic flux [116]. In fact, it has been shown that cytoplasmic p53 inhibits autophagy [117]. However, nuclear p53 activates the autophagic pathway [116]. These data confirm our findings implicating that autophagic flux execution upon NP treatment is impaired in Alexander cells.

**Figure 9:**
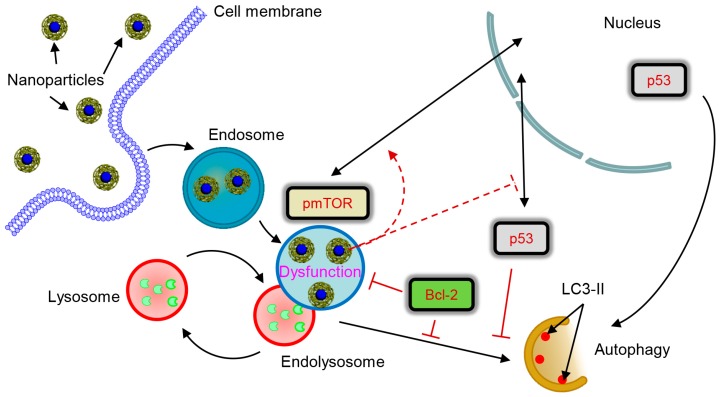
Scheme of lysosomal dysfunction upon nanoparticle treatment. Sub-lethal doses of iron oxide nanoparticles are endocytosed into lysosomes in hepatic cells within 12 h. Accumulation of nanoparticles in lysosomal compartments leads to progressive impairment of lysosomal function. The resulted lysosomal dysfunction probably affects sub-cellular localization of pmTOR and p53. Progressive lysosomal dysfunction leads to initiation of autophagic flux, which is supported by nuclear p53. Contrary cytosolic p53 and high levels of Bcl-2 inhibit autophagy.

We can propose a tentative signaling scheme of NP-hepatic cell interactions (Figure 9). Altogether, our data imply that the control hub of nanoparticle-mediated responses within the cell is the lysosome. Logically, the expression of proteins that regulate lysosomal function (e.g. Bcl-2 and p53) greatly contributes to the cellular response to NPs. Indeed, the genetic background of the cells predisposes their reaction to NPs. One has to take into account levels of Bcl-2 expression when targeting lysosomes with NPs to induce apoptosis or autophagy. It is worth noting here that, in this study, we used liver tumour cell lines. Further research is needed to assess how autophagic flux would be executed in non-cancer cells. Our results are of great importance for the future development of targeted nanoparticle-based therapies of various pathological conditions.

## Supplementary Materials

The following are available online at https://www.mdpi.com/2073-4409/9/4/1015/s1, Table S1: Chemicals and fluorescent probes used in the study, Table S2: Antibodies used in the study, Figure S1: Physicochemical characterization of fluorescent (NPs-Fl) and non-fluorescent (NPs) carboxymethyldextran-coated iron oxide nanoparticles, Figure S2: Nanoparticle−protein interaction, Figure S3: NP treatment did not induce intracellular ROS/Superoxide (O_2_^−^) production and different subcellular accumulation, Figure S4: Cytoskeleton remodeling under NP treatment, Figure S5: Localization of fluorescently labeled nanoparticles (green) in lysosomal compartments, Figure S6: Lysosomal integrity as measured by acridine orange (AO) red fluorescence decrease, Figure S7: Densitometric quantification of blots represented in Figure 5g, Figure S8: Assessment of mitochondria integrity and induction of mitochondrial membrane depolarization by NP treatment, Figure S9: Colocalization analysis of nanoparticles and Rab7 protein, Figure S10: Confirmation of autophagic flux by formation of cellular autophagosome punctae containing LC3-II, Figure S11: Confirmation of autophagic flux induced by NP in Huh7 cells, Figure S12: Sub-cellular localization of pmTOR upon nanoparticle treatment, Figure S13: Representative confocal microscopic images of p53 sub-cellular localization in distinct cell lines, Figure S14: Bcl-2 was analyzed in whole cell lysates of HepG2, Huh7 and Alexander cells by immunoblotting, Figure S15: Representative confocal microscopic images of p53 sub-cellular localization in distinct cell lines upon nanoparticle treatment, Uncropped immunoblot scans.
