# Supplementary material for: Iron Oxide Nanoparticle-Induced Autophagic Flux Is Regulated by Interplay between p53-mTOR Axis and Bcl-2 Signaling in Hepatic Cells
Source: Cells. 2020 Apr 18;9(4):1015. doi: 10.3390/cells9041015 (PMC7226334; doi:10.3390/cells9041015)

Supplementary material to

Iron Oxide Nanoparticle-induced Autophagic Flux is Regulated by Interplay between p53-mTOR Axis and Bcl-2 Signaling in Hepatic Cells

Mariia Uzhytchak [1,†], Barbora Smolková [1,†], Mariia Lunova [1,2], Milan Jirsa [2], Adam Frtús [1], Šárka Kubinová [1,3], Alexandr Dejneka [1] and Oleg Lunov [1,*]

[1] Institute of Physics of the Czech Academy of Sciences, Prague, 18221, Czech Republic; uzhytchak@fzu.cz (M.U.); smolkova@fzu.cz (B.S.); frtus@fzu.cz (A.F.); dejneka@fzu.cz (A.D.); lunov@fzu.cz (O.L.)
[2] Institute for Clinical & Experimental Medicine (IKEM), Prague, 14021, Czech Republic; mariialunova@googlemail.com (M.L.); miji@ikem.cz (M.J.)
[3] Institute of Experimental Medicine of the Czech Academy of Sciences, Prague, 14220, Czech Republic; sarka.kubinova@iem.cas.cz (S.K.)
[*] Correspondence: lunov@fzu.cz; Tel.: +420266052131
[†] These authors contributed equally to this work.

Supplementary Tables

Table S1 Chemicals and fluorescent probes used in the study.

Reagent	Manufacturer	Catalogue
AlamarBlue	ThermoFisher Scientific	DAL 1025
Trypan Blue	ThermoFisher Scientific	15250061
BODIPY™ 581/591 C11	ThermoFisher Scientific	D3861
Cell culture media Eagle's Minimum Essential Medium (EMEM)	ATCC	ATCC® 30-2003™
Fluorescent nanoparticles nano-screenMAG-CMX	Chemicell	4406-5
Non-fluorescent nanoparticles fluidMAG-CMX	Chemicell	4106-5
Acridine Orange	ThermoFisher Scientific	A3568
Paraformaldehyde	VWR	100503-917
PBS	Gibco	10010015
Triton-X100	PanReac AppliChem	A4975,0100
Micro BCA Protein Assay Kit	Thermo Fisher Scientific	23235
RIPA buffer	Millipore	20188
Hoechst 33342	Thermo Fisher Scientific	62249
NE-PER Nuclear and Cytoplasmic Extraction Kit	Thermo Fisher Scientific	78835
Protease Inhibitor Cocktail	Sigma Aldrich	P8340-1ML
Phosphatase Inhibitor Cocktail 3	Sigma Aldrich	P0044-1ML
LysoTracker™ Red DND-99	Thermo Fisher Scientific	L12491
CellMask™ Orange	ThermoFisher Scientific	C10045
Cellular ROS/Superoxide Detection Assay Kit	Abcam	ab139476
Coomassie Brilliant blue R-250	AppliChem	A1092
Bafilomycin A_1	Sigma	SML1661

Table S2 Antibodies used in the study.

Antibody	Clone/catalogue number	Manufacturer	Dilution	
			WB	IF
Anti-Rab7	D95F2/9367	Cell Signaling Technology	1:1000	1:100
Anti-Cathepsin B	D1C7Y/21718	Cell Signaling Technology	1:1000	NA
Anti-HDAC2	N.A./2540	Cell Signaling Technology	1:1000	NA
Anti-LC3A/B	D3U4C/12741	Cell Signaling Technology	1:1000	1:100
Anti-mTOR	L27D4/4517	Cell Signaling Technology	1:1000	1:100
Anti-P53	7F5/2527	Cell Signaling Technology	1:1000	1:1000
Anti-pmTOR	Ser2448/2971S	Cell Signaling Technology	1:1000	1:100
Anti-LAMP1	D401S/15665	Cell Signaling Technology	1:1000/1:100	1:100
Anti- β-Tubulin	D2N5G/ 15115	Cell Signaling Technology	1:1000	1:100
F-actin ActinGreen™ 488 ReadyProbes™ Reagent	R37110	Thermo Fisher Scientific	NA	2 drops per mL of medium

Anti-β-actin	10D10	Thermo Fisher Scientific	1:1000	NA
Anti-Bcl-2	15071	Cell Signaling Technology	1:1000	NA
Anti-Mouse-HRP	G21040	Thermo Fisher Scientific	1:10000	NA
Anti-Rabbit-HRP	G21234	Thermo Fisher Scientific	1:10000	NA
Anti- mouse-HRP	1858413	Pierce Biotechnology	1:10000	NA
Anti-rabbit- HRP	1858415	Pierce Biotechnology	1:10000	NA
AlexaFluor 568 goat anti-rabbit IgG	A-11011	Thermo Fisher Scientific	NA	1:1000
AlexaFluor 568, goat anti-mouse antibody IgG	A-11004	Thermo Fisher Scientific	NA	1:1000
AlexaFluor 488 goat anti-rabbit antibody IgG	A-11008	Thermo Fisher Scientific	NA	1:1000

NA – not available; WB – western blot; IF – immunofluorescence.

Supplementary Figures

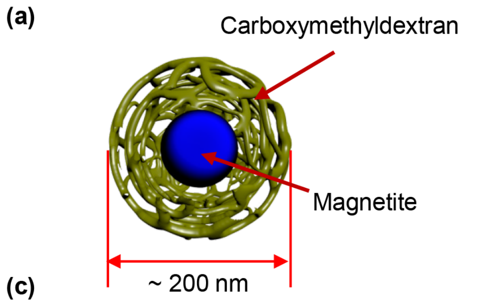

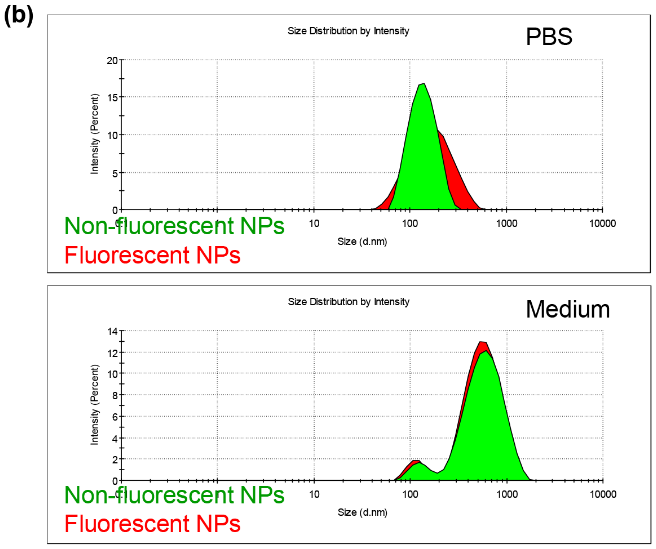

(c)

Particle	ξ (mV)	PDI	Hydrodynamic diameter (nm)
NPs PBS	-1.76	0.078	206
NPs-Fl PBS	-1.82	0.109	210
NPs Medium	-15.02	0.244	437
NPs-Fl Medium	-16.31	0.256	454

Figure S1. Physicochemical characterization of fluorescent (NPs-Fl) and non-fluorescent (NPs) carboxymethyldextran-coated iron oxide nanoparticles. (**a**) Scheme of nanoparticle structure. (**b**) Hydrodynamic diameters of fluorescent (NPs-Fl) and non-fluorescent (NPs) nanoparticles as measured by laser light scattering. Particles were dissolved either in PBS or cell culture medium. (**c**) Surface characterization of the particles dissolved either in PBS or cell culture mediumm measured with a Zetasizer Nano (PDI – polydispersity index; ζ – zeta potential).

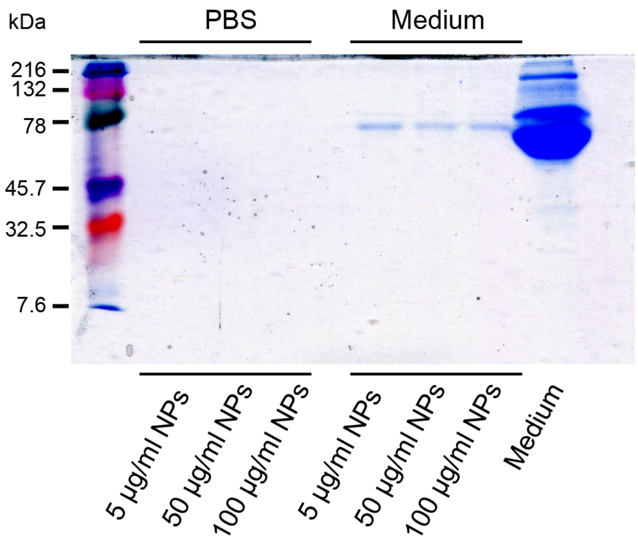

Figure S2. Nanoparticle–protein interaction. Nanoparticles (10, 50 and 100 µg Fe mL^{-1}) were incubated either in PBS, or in EMEM medium (ATCC) supplemented with 10% fetal bovine serum (FBS, Thermo Fisher Scientific) for 2 h at 37 °C. The particles were collected by strong NdFeB magnet and washed extensively with PBS. The proteins associated with the particles were eluted and denaturated in sample loading buffer and separated by gel electrophoresis. Gels were stained with Coomassie blue (AppliChem).

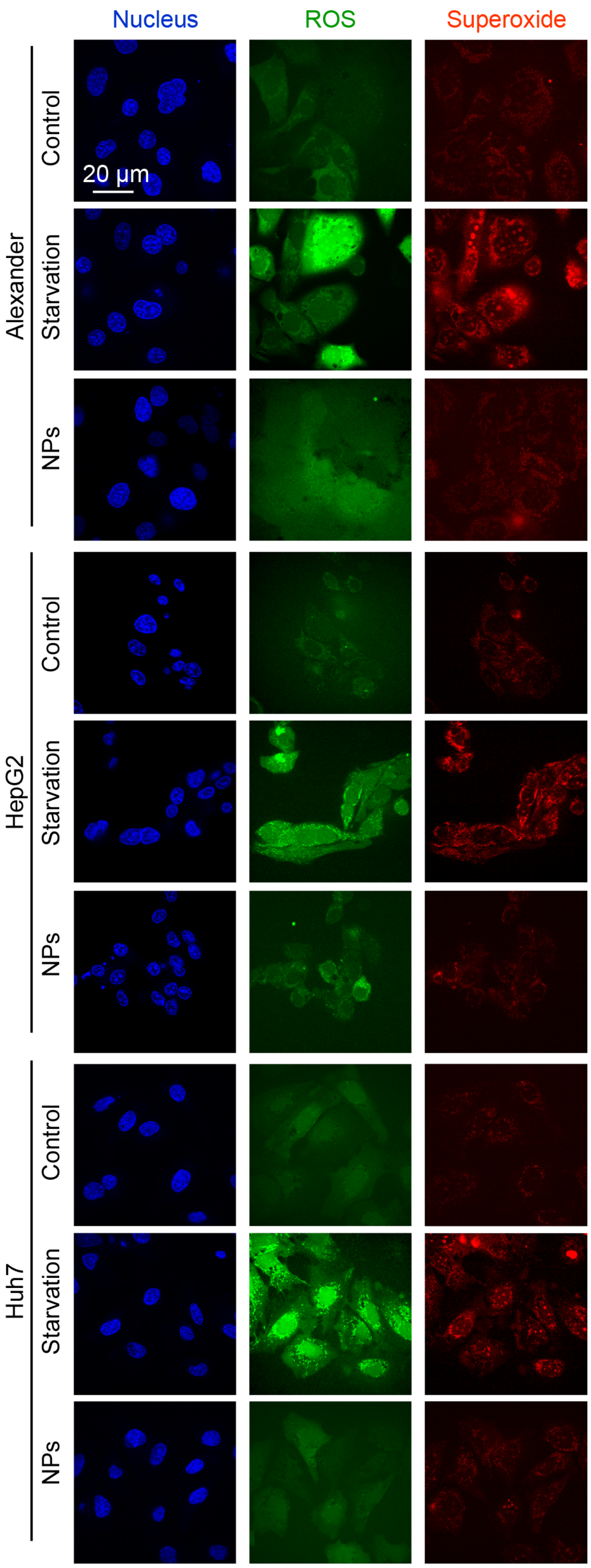

Figure S3. NP treatment did not induce intracellular ROS / Superoxide (O_2^-) production and different subcellular accumulation. Cells were treated for 24 h with nanoparticles 50 μg Fe

mL^{-1}. NP-treated cells were stained with ROS/Superoxide Detection Assay Kit and imaged by confocal microscopy. Representative images out of three independent experiments are shown. Positive control 1 mM H_2O_2 for 30 min was used.

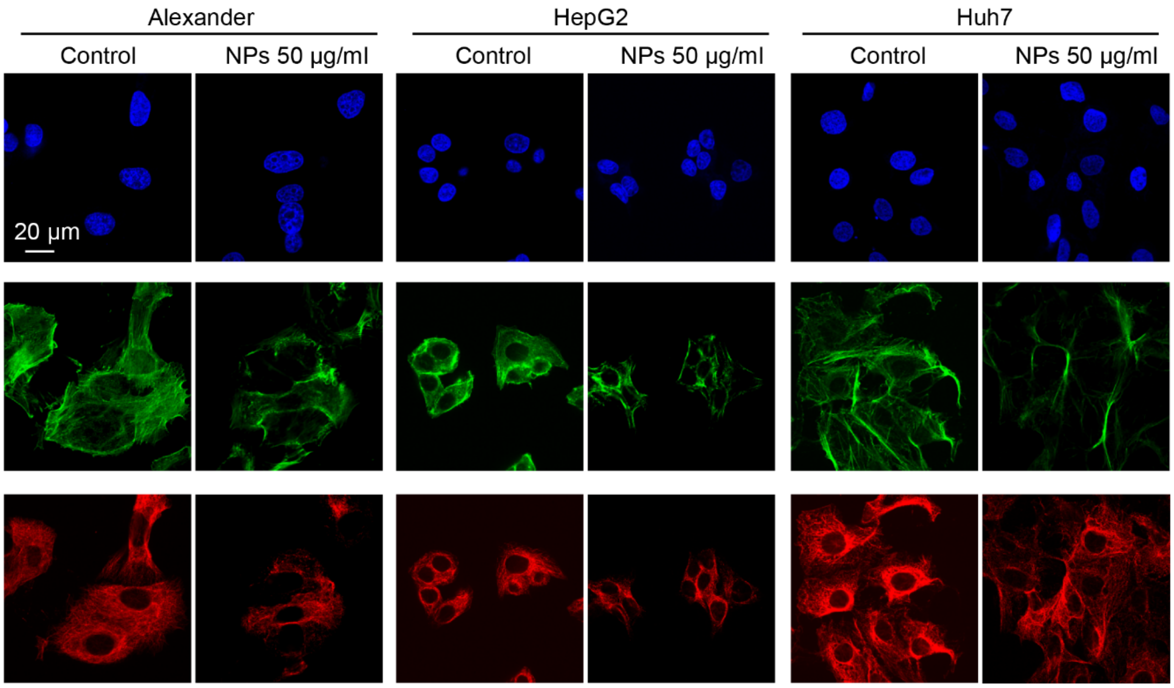

Figure S4. Cytoskeleton remodeling under NP treatment. Cells were treated for 24 h with nanoparticles 50 µg Fe mL^{-1}, fixed and stained for F-actin (green) and tubulin (red). Nuclei were stained with hoechst 33342 nuclear stain (blue). Labeled cells were then imaged using spinning disk confocal microscopy.

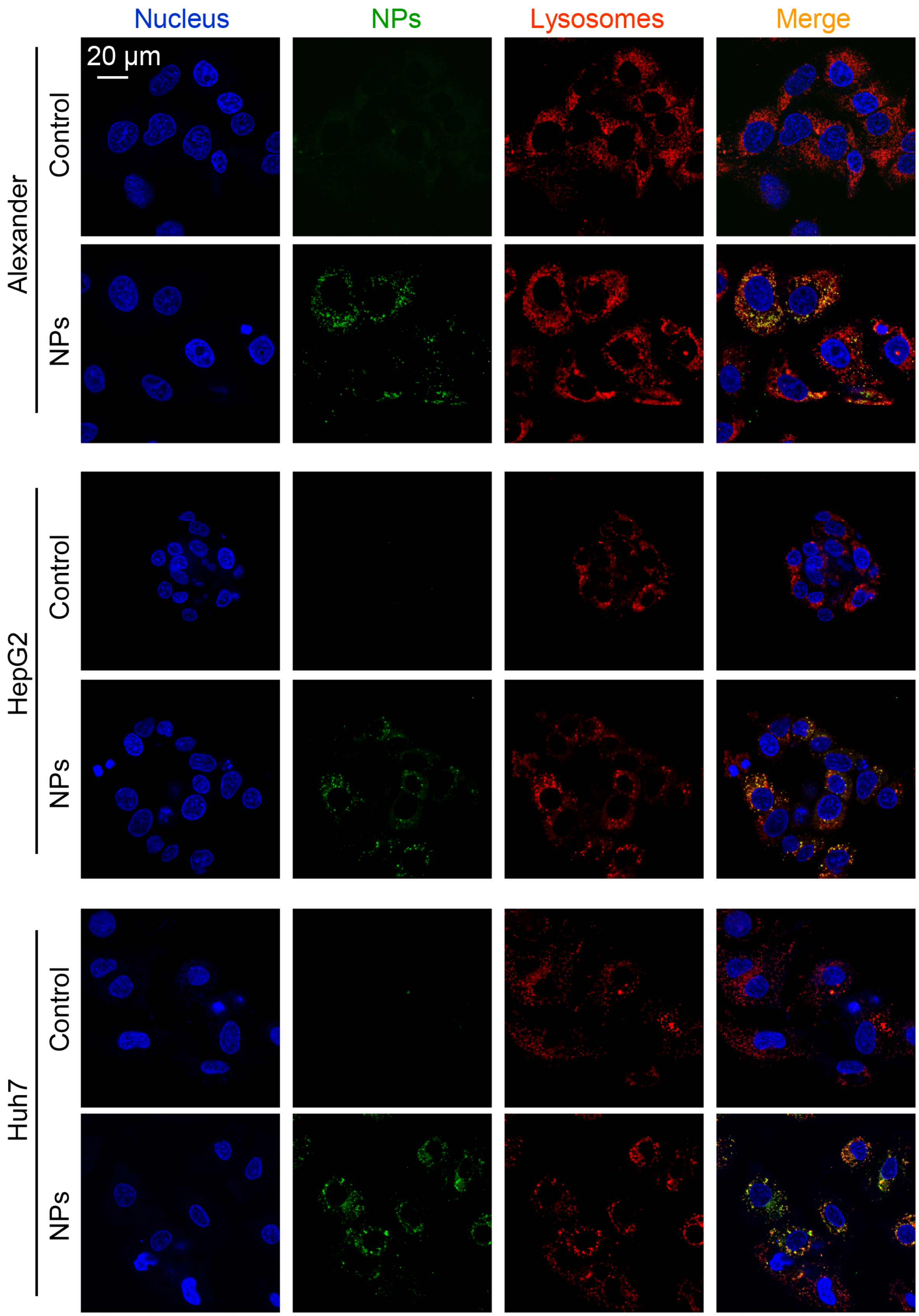

Figure S5. Localization of fluorescently labeled nanoparticles (green) in lysosomal compartments. Cells were treated for 12 h with nanoparticles 50 µg Fe mL^{-1} and labeled with LysoTracker™ Red DND-99 (red). Nuclei were stained with hoechst 33342 nuclear stain

(blue). Merge of green and red gives yellow color. Labeled cells were then imaged using spinning disk confocal microscopy.

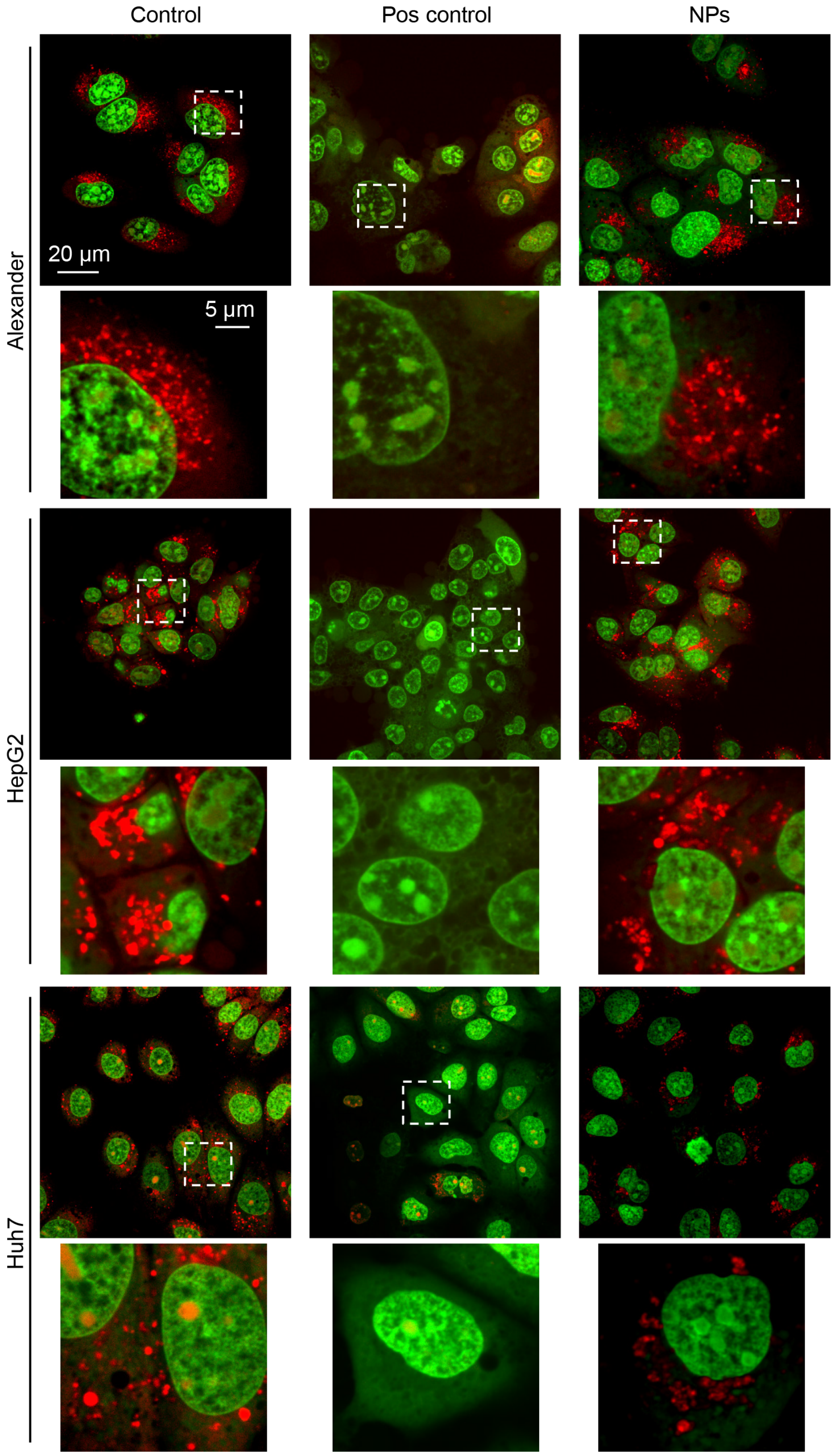

Figure S6. Lysosomal integrity as measured by acridine orange (AO) red fluorescence decrease. Cells were treated with 100 μg Fe mL^{-1} nanoparticles for 24 h, stained with AO and then imaged using spinning disk confocal microscopy. Positive control – 20 % ethanol for 10 min.

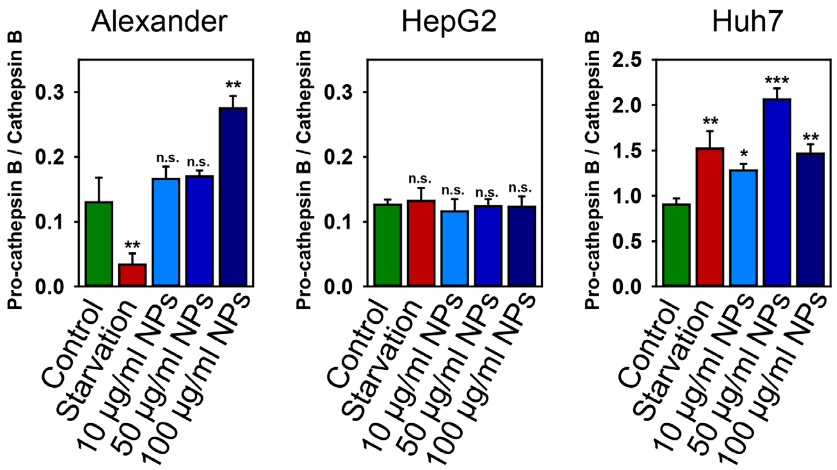

Figure S7. Densitometric quantification of blots represented in Figure 5g.

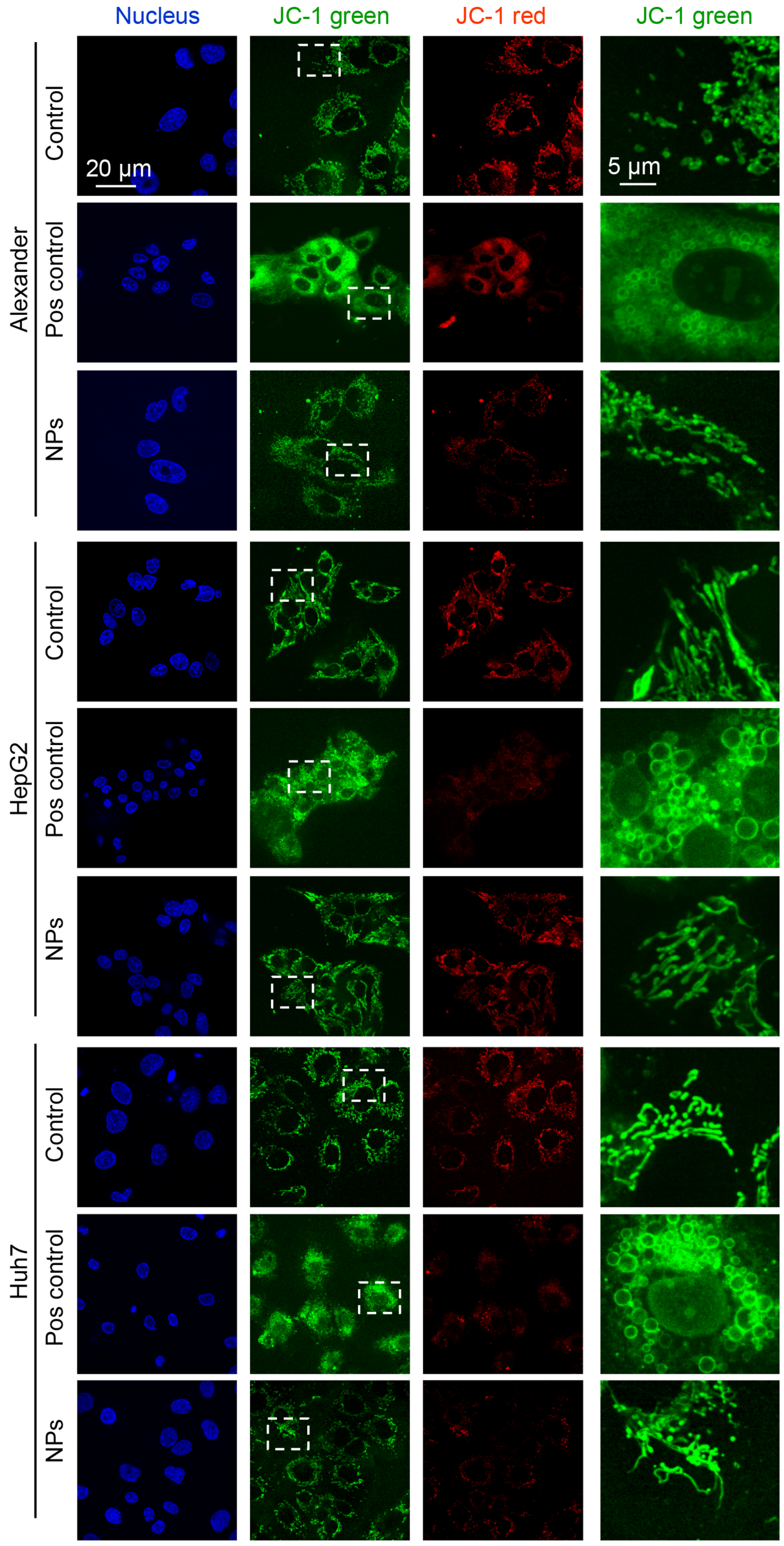

Figure S8. Assessment of mitochondria integrity and induction of mitochondrial membrane depolarization by NP treatment. Cells were treated with 100 µg Fe mL^{-1} nanoparticles for 24

h, stained with JC-1 (1 μM) and then imaged using spinning disk confocal microscopy. Positive control – 20 % ethanol for 30 min.

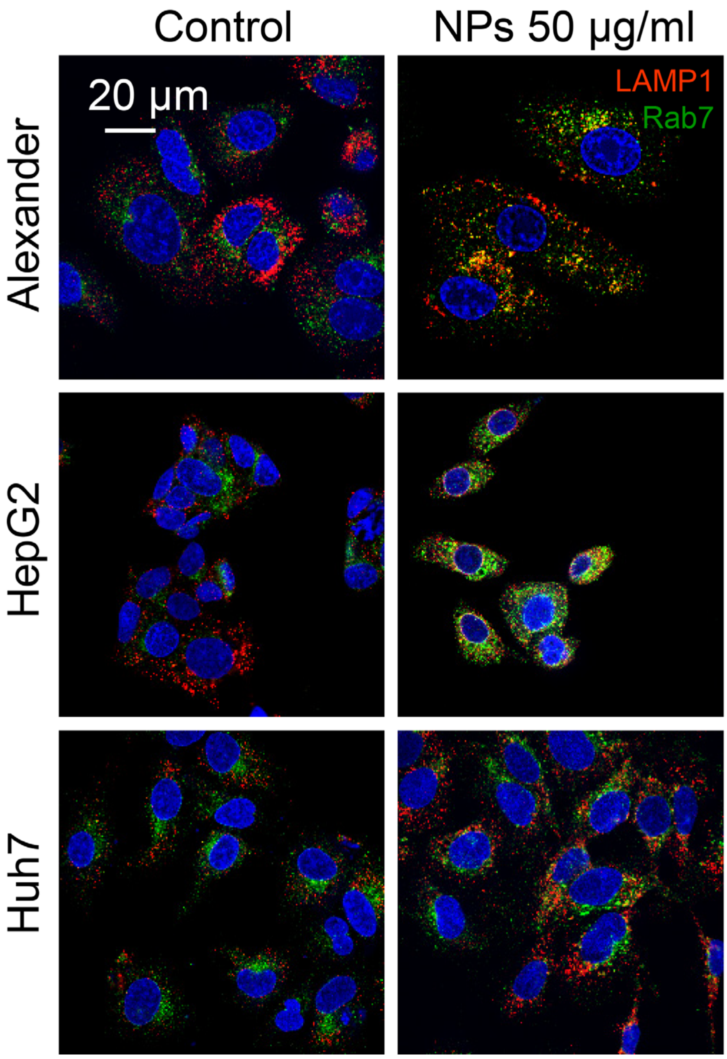

Figure S9. Colocalization analysis of nanoparticles and Rab7 protein. Cells were treated for 12 h with nanoparticles 50 μg Fe mL^{-1}, fixed and immunostained for LAMP1 (red) and Rab7 (green). Labeled cells were then imaged using spinning disk confocal microscopy.

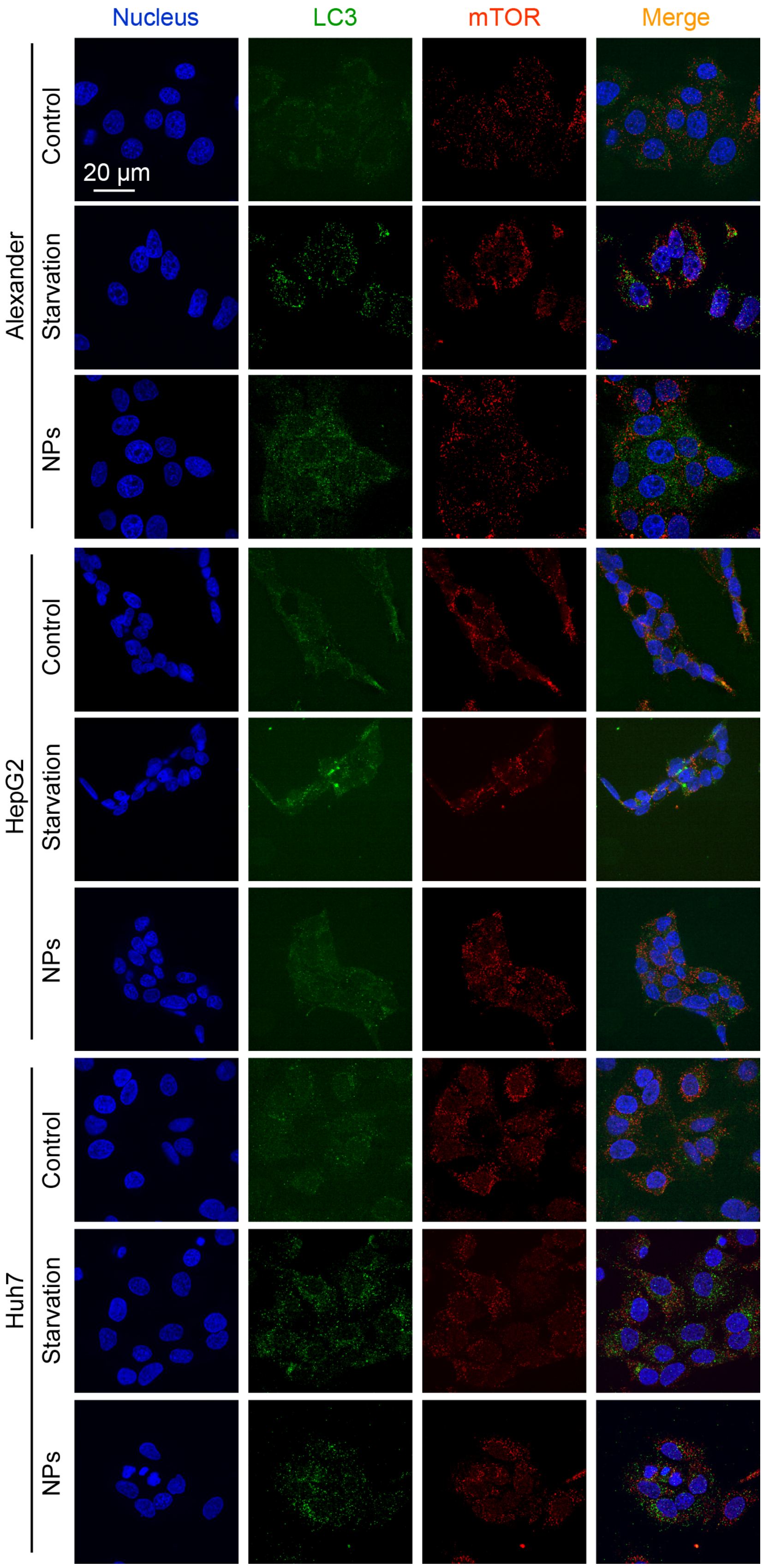

Figure S10. Confirmation of autophagic flux by formation of cellular autophagosome punctae containing LC3-II. Cells were treated for 12 h with nanoparticles 50 μg Fe mL^{-1}, fixed and immunostained for mTOR (red) and LC3 (green). Labeled cells were then imaged

using spinning disk confocal microscopy. Positive control – serum starvation for 12 (Alexander, Huh7) and 14 (HepG2) h. Nuclei were stained with Hoechst 33342.

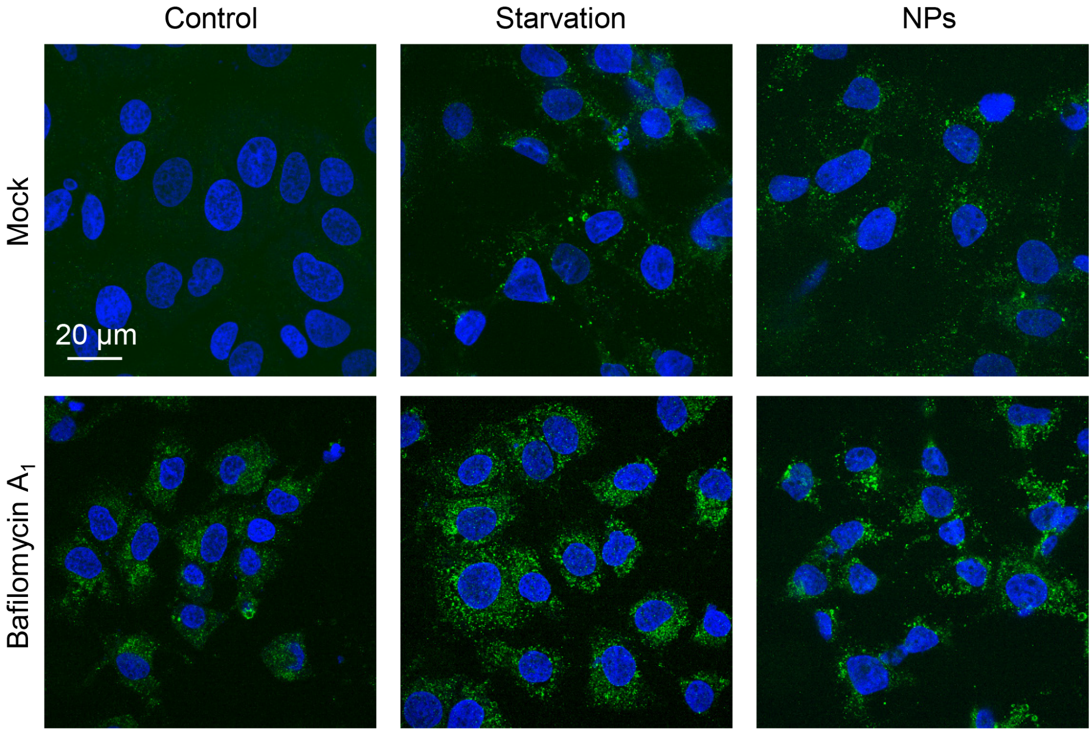

Figure S11. Confirmation of autophagic flux induced by NP in Huh7 cells. Cells were treated for 24 h with nanoparticles 100 µg Fe mL^{-1} in the presence or absence of bafilomycin A_1 (100 nM), fixed and immunostained for LC3 (green). Labeled cells were then imaged using spinning disk confocal microscopy. Positive control – serum starvation for 12 h. Nuclei were stained with Hoechst 33342.

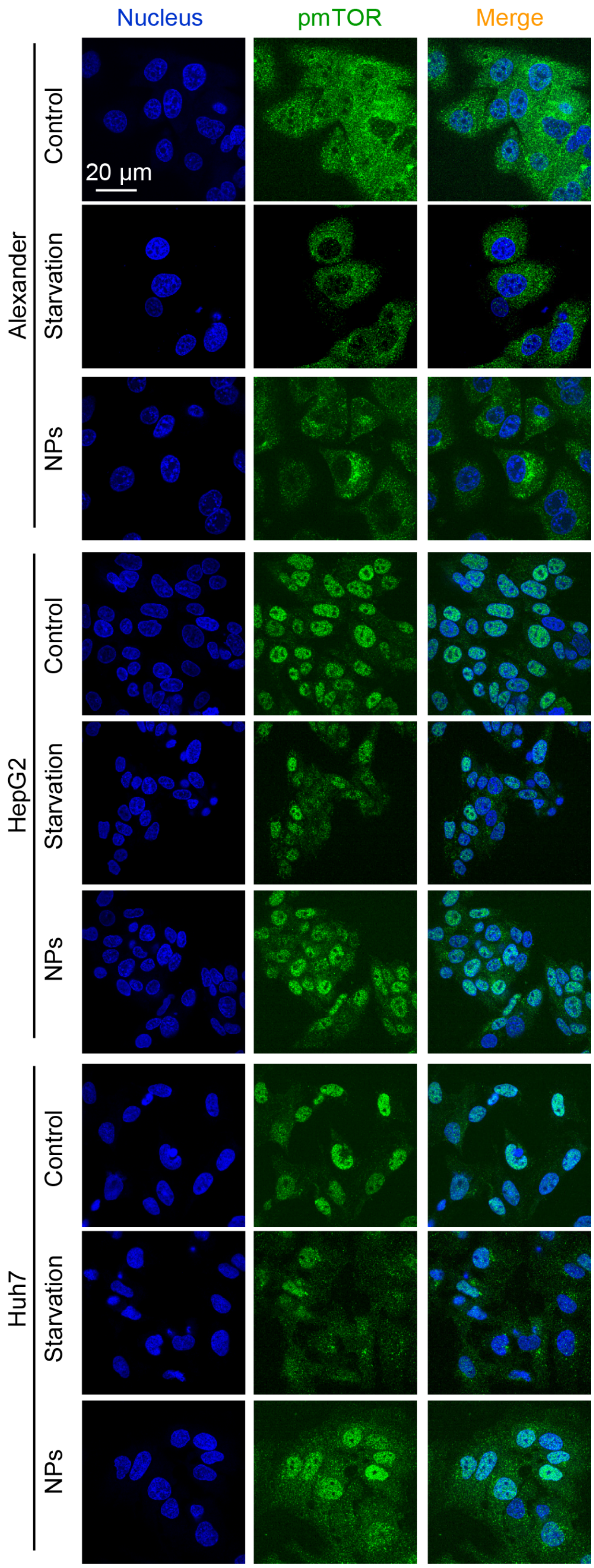

Figure S12. Sub-cellular localization of pmTOR upon nanoparticle treatment. Representative confocal microscopic images of three cell lines. Cells were treated for 12 h with nanoparticles 50 μg Fe mL^{-1}, fixed and immunostained for pmTOR (green). Positive control – serum starvation for 12 (Alexander, Huh7) and 14 (HepG2) h. Nuclei were stained with Hoechst 33342.

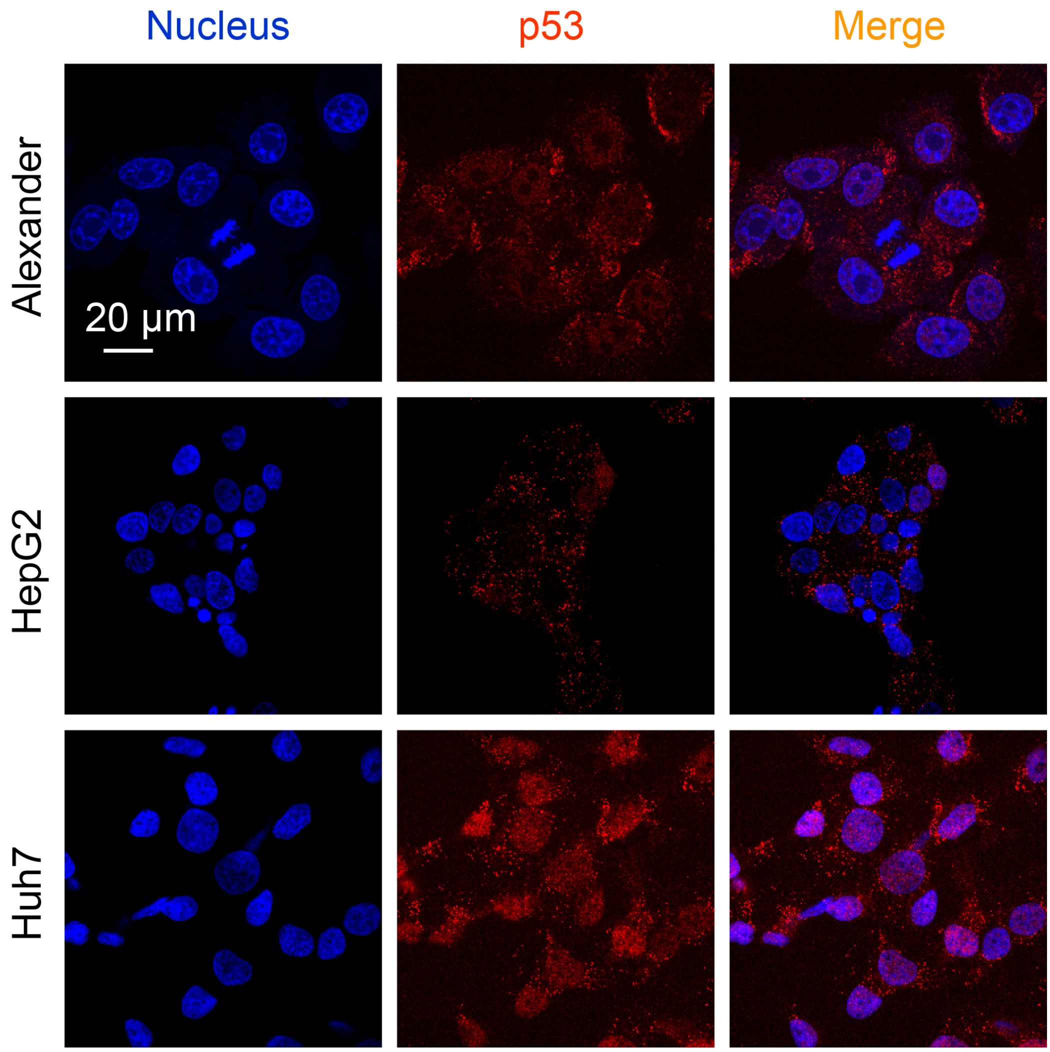

Figure S13. Representative confocal microscopic images of p53 sub-cellular localization in distinct cell lines. Huh7, HepG2 and Alexander cells were fixed and immunostained for p53 (red). Nuclei were stained with Hoechst 33342. Labeled cells were then imaged using spinning disk confocal microscopy.

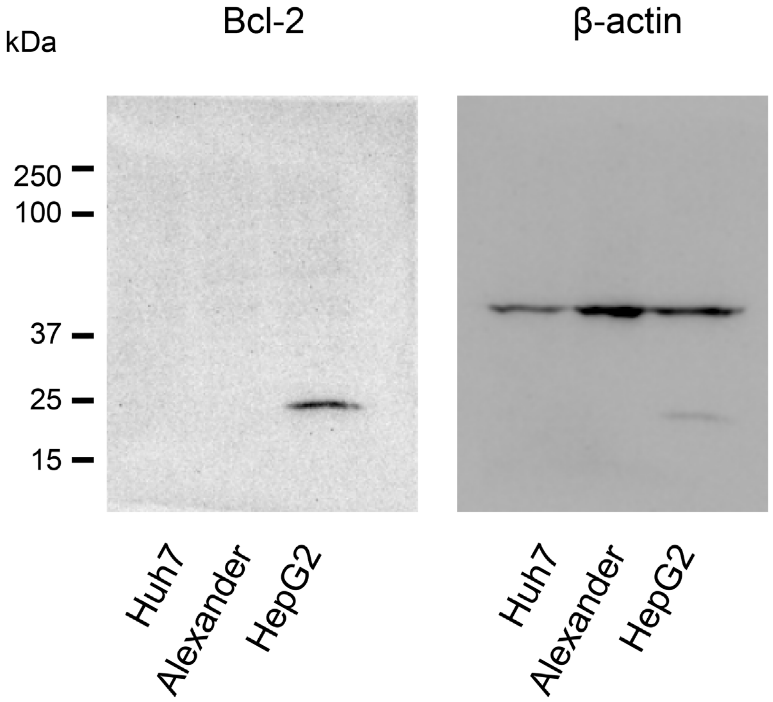

Figure S14. Bcl-2 was analyzed in whole cell lysates of HepG2, Huh7 and Alexander cells by immunoblotting; Actin – control of equal protein loading.

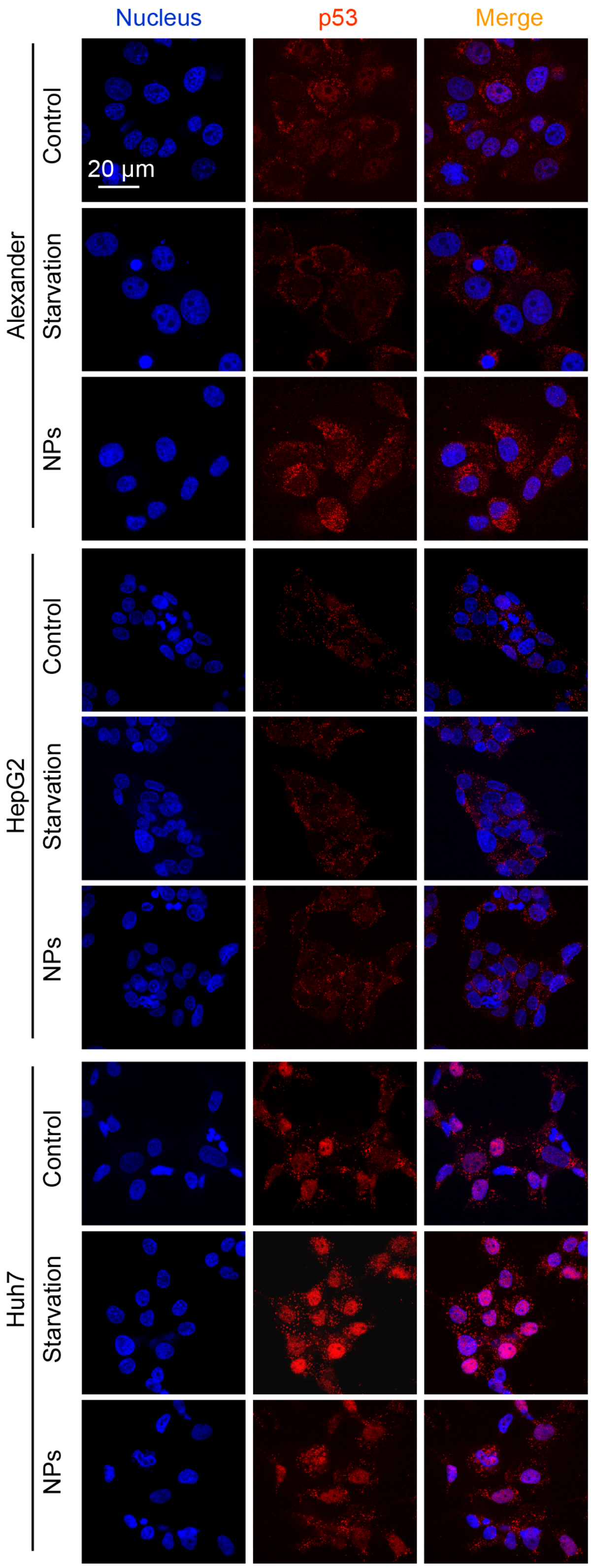

Figure S15. Representative confocal microscopic images of p53 sub-cellular localization in distinct cell lines upon nanoparticle treatment. Cells were treated for 12 h with nanoparticles 50 µg Fe mL^{-1}, fixed and immunostained for p53 (red). Labeled cells were then imaged using spinning disk confocal microscopy. Positive control – serum starvation for 12 h. Nuclei were stained with Hoechst 33342.

Uncropped immunoblot scans

Figure 5g

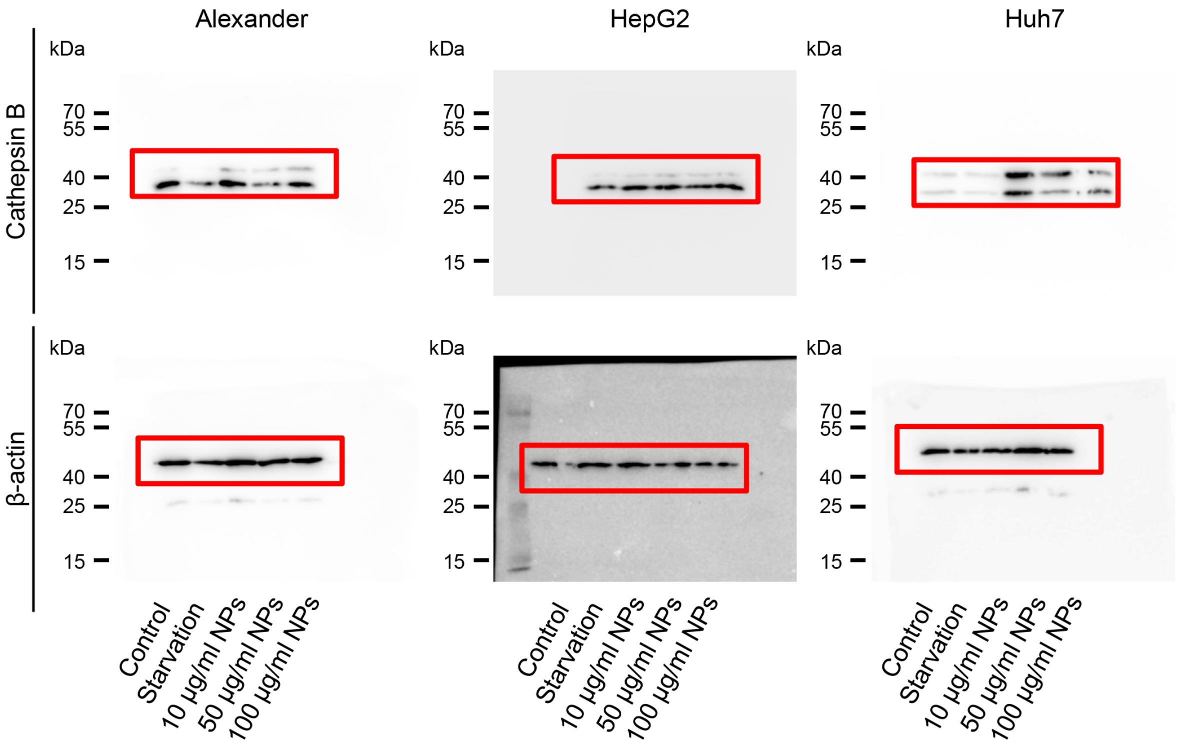

Figure 6b

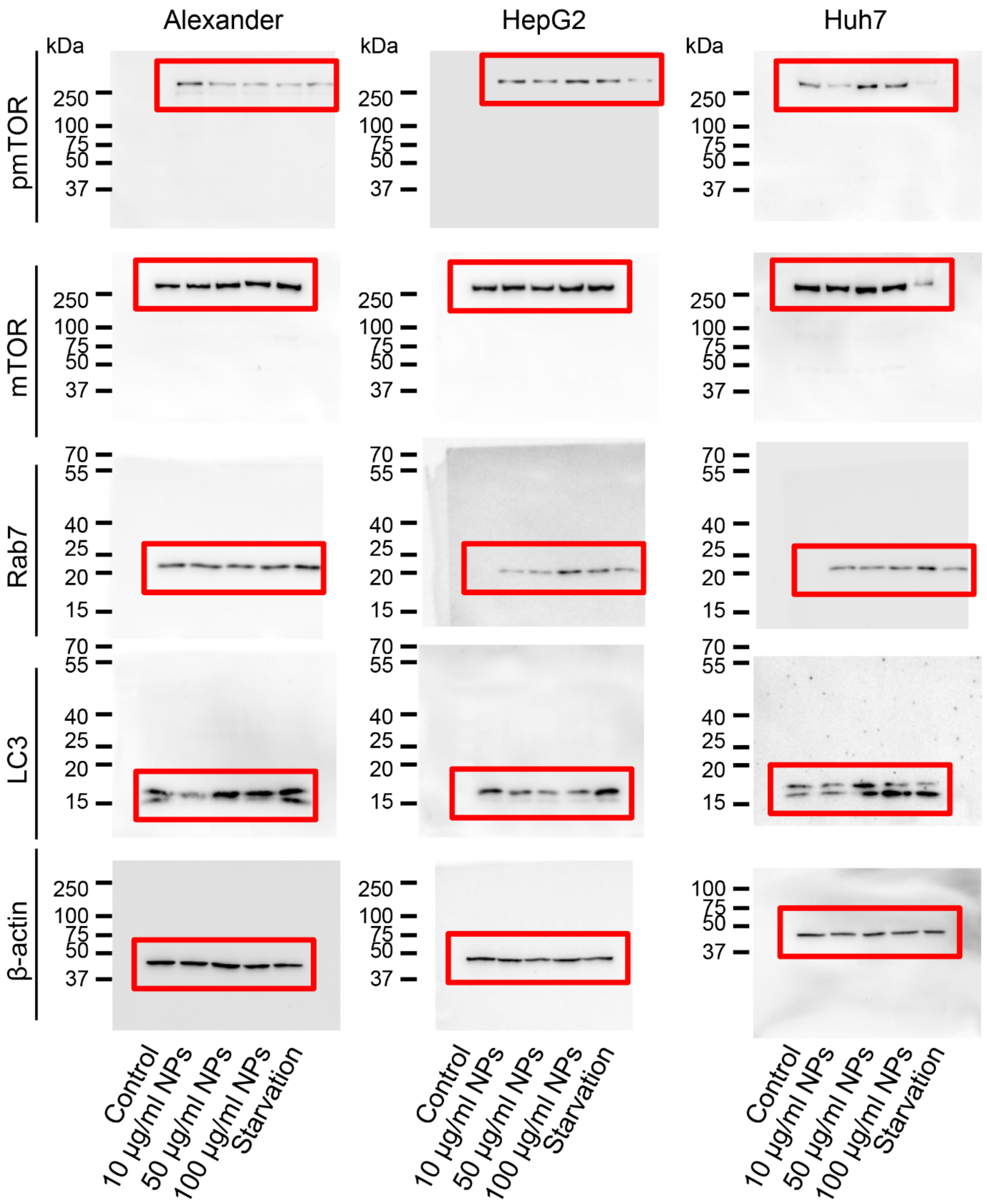

Alexander
HepG2
Huh7
kDa
pmTOR
mTOR
Rab7
LC3
β-actin
250
100
75
50
37
70
55
40
25
20
15
Control
10 µg/ml NPs
50 µg/ml NPs
100 µg/ml NPs
Starvation

Figure 7b

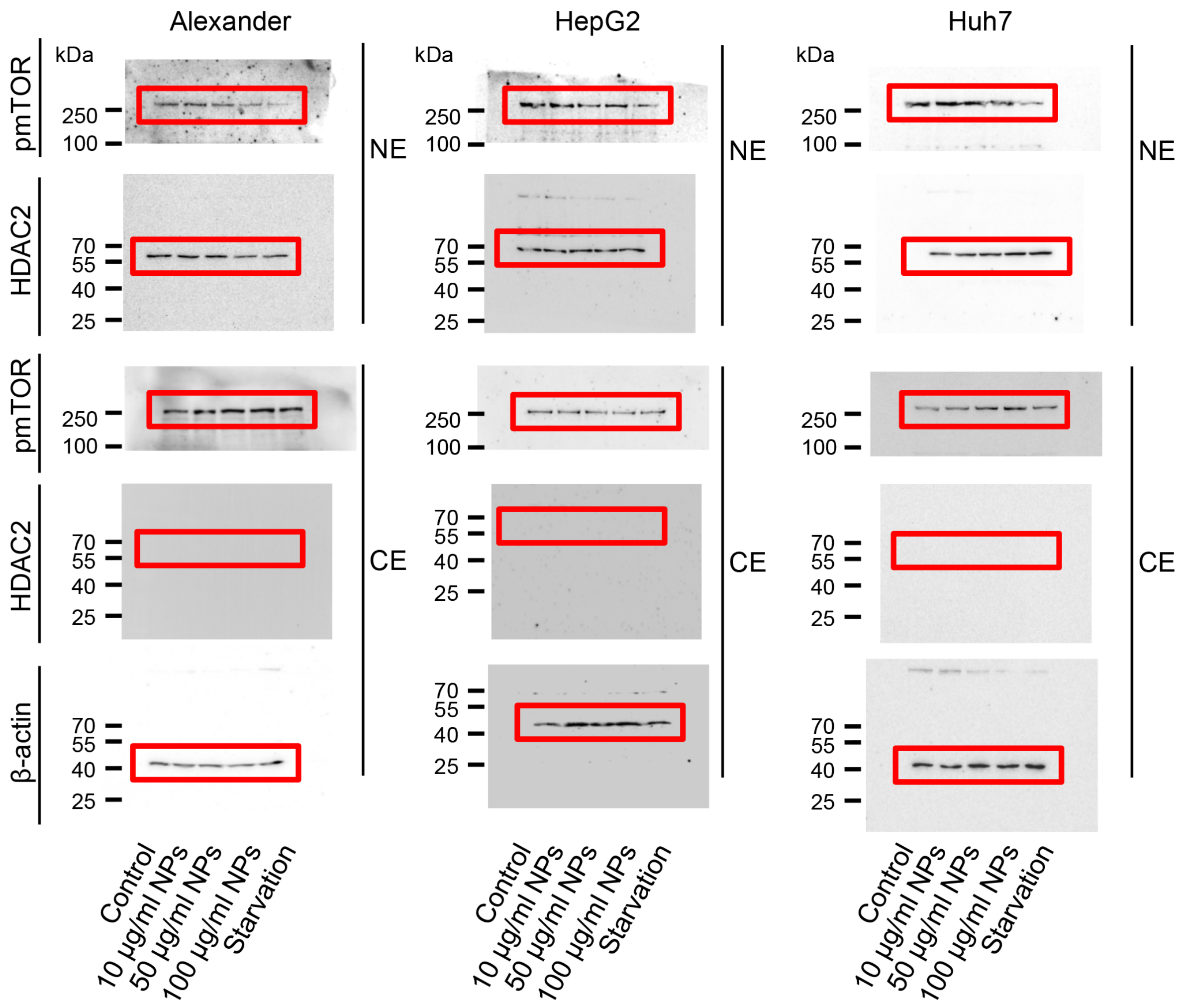
Alexander
HepG2
Huh7
kDa
pmTOR
HDAC2
β-actin
NE
CE
250
100
70
55
40
25
Control
10 µg/ml NPs
50 µg/ml NPs
100 µg/ml NPs
Starvation

Figure 8c

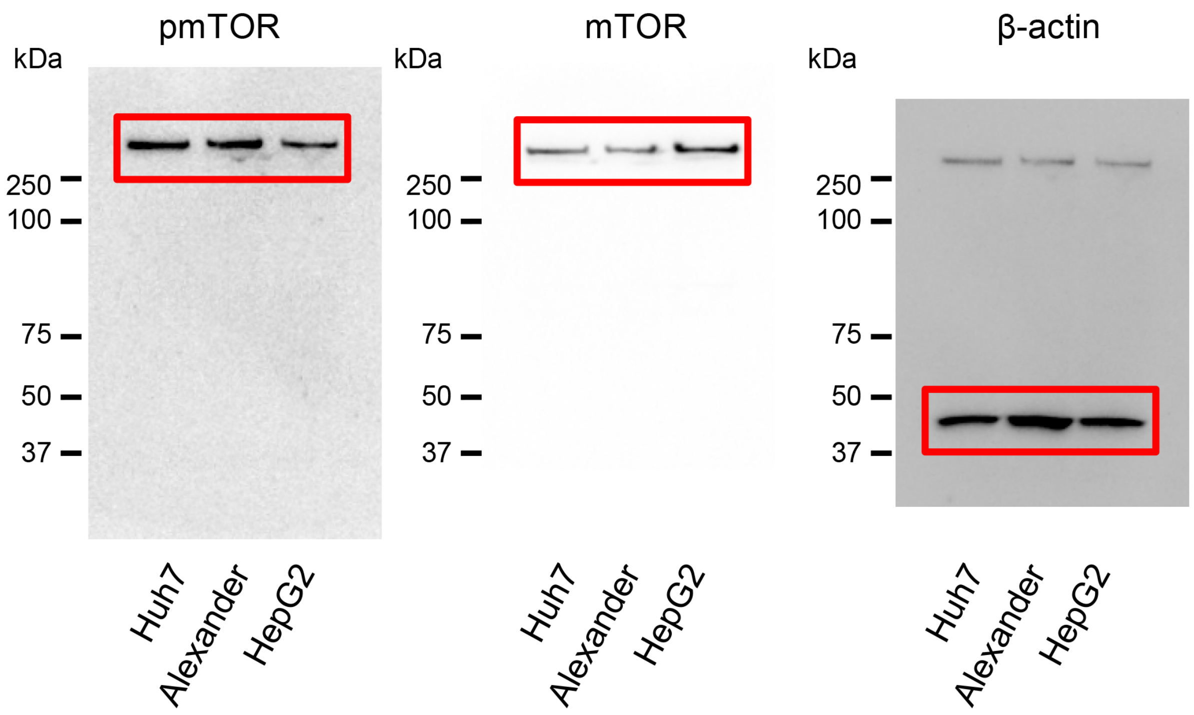

Supplement: Supplementary file 1 [file cells-09-01015-s001.pdf]
